# Suppression of food intake by *Glp1r/Lepr*-coexpressing neurons prevents obesity in mouse models

**DOI:** 10.1172/JCI157515

**Published:** 2023-10-02

**Authors:** Alan C. Rupp, Abigail J. Tomlinson, Alison H. Affinati, Warren T. Yacawych, Allison M. Duensing, Cadence True, Sarah R. Lindsley, Melissa A. Kirigiti, Alexander MacKenzie, Joseph Polex-Wolf, Chien Li, Lotte Bjerre Knudsen, Randy J. Seeley, David P. Olson, Paul Kievit, Martin G. Myers

**Affiliations:** 1 Department of Internal Medicine and; 2 Department of Molecular and Integrative Physiology, University of Michigan, Ann Arbor, Michigan, USA.; 3 Oregon National Primate Research Center, Beaverton, Oregon, USA.; 4 Novo Nordisk, Copenhagen, Denmark.; 5 Department of Surgery and; 6 Department of Pediatrics, University of Michigan, Ann Arbor, Michigan, USA.

**Keywords:** Endocrinology, Metabolism, Leptin, Mouse models, Obesity

## Abstract

The adipose-derived hormone leptin acts via its receptor (LepRb) in the brain to control energy balance. A potentially unidentified population of GABAergic hypothalamic LepRb neurons plays key roles in the restraint of food intake and body weight by leptin. To identify markers for candidate populations of LepRb neurons in an unbiased manner, we performed single-nucleus RNA-Seq of enriched mouse hypothalamic LepRb cells, identifying several previously unrecognized populations of hypothalamic LepRb neurons. Many of these populations displayed strong conservation across species, including GABAergic *Glp1r*-expressing LepRb (LepRb^Glp1r^) neurons, which expressed more *Lepr* than other LepRb cell populations. Ablating *Lepr* from LepRb^Glp1r^ cells provoked hyperphagic obesity without impairing energy expenditure. Similarly, improvements in energy balance caused by *Lepr* reactivation in GABA neurons of otherwise *Lepr*-null mice required *Lepr* expression in GABAergic *Glp1r*-expressing neurons. Furthermore, restoration of *Glp1r* expression in LepRb^Glp1r^ neurons in otherwise *Glp1r*-null mice enabled food intake suppression by the GLP1R agonist, liraglutide. Thus, the conserved GABAergic LepRb^Glp1r^ neuron population plays crucial roles in the suppression of food intake by leptin and GLP1R agonists.

## Graphical abstract

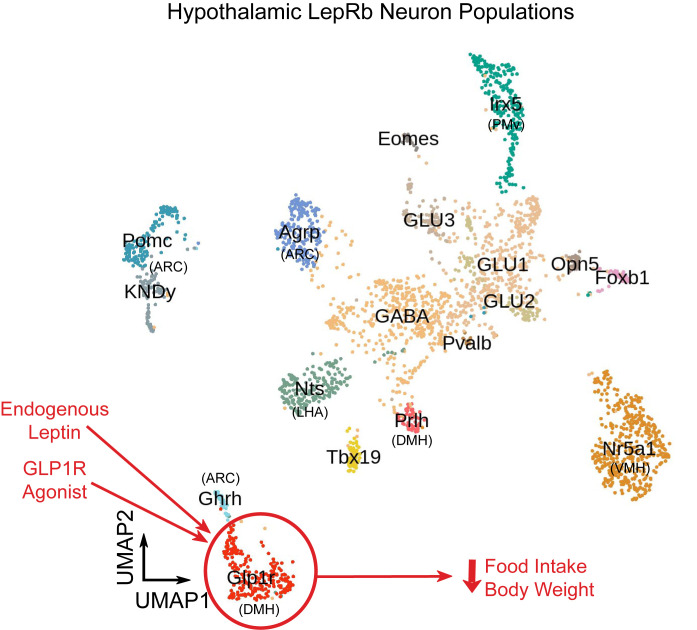

## Introduction

The ongoing obesity pandemic represents an enormous challenge to human health and longevity (1). Identifying therapeutic targets to combat obesity will require understanding the physiologic systems that modulate feeding and maintain appropriate body weight. The adipose-derived hormone, leptin, controls body weight by signaling the repletion of body fat stores to hypothalamic neurons that express the long isoform of the leptin receptor (LepRb) (2, 3). Insufficient leptin action, as following the reduction of fat stores by prolonged caloric restriction, increases hunger and inhibits energy-intensive neuroendocrine systems (4, 5). Similarly, humans and animals lacking leptin or LepRb are unable to sense adipose energy stores and thus exhibit voracious feeding and decreased energy expenditure despite their severe obesity.

Hypothalamic LepRb neurons that control energy balance represent important potential targets for obesity therapy, but the cell types most important for the control of food intake and body weight by direct leptin action remain to be defined. Although arcuate nucleus (ARC) proopiomelanocortin-expressing (*Pomc*-expressing) and distinct agouti-related peptide–expressing (*Agrp*-expressing) neurons play important roles in energy balance (6, 7), the early developmental ablation of *Lepr* from these cells only modestly affects feeding and body weight (8–10). While developmental compensation may partially conceal the role for LepRb in AgRP neurons following its early ablation (9, 11, 12), animals lacking LepRb from early developmental times (throughout the body or the hypothalamus) exhibit dramatic hyperphagic obesity despite any compensation for their lack of LepRb in AgRP neurons or elsewhere (13). Thus, other (non-AgRP) hypothalamic LepRb neurons not subject to the same level of developmental compensation as AgRP neurons must play major roles in leptin action.

Several lines of evidence suggest the importance of an as-yet unidentified set of GABAergic LepRb neurons for the control of food intake and body weight; these likely reside in the dorsomedial hypothalamic nucleus (DMH) (10, 14–16). To identify potentially important hypothalamic LepRb neuron populations, we utilized single-nucleus RNA-Seq (snRNA-Seq) to define hypothalamic LepRb neuron populations in an unbiased manner. In addition to identifying previously described types of LepRb neurons, this analysis revealed several previously unknown LepRb populations, including those marked by *Glp1r*, *Tbx19*, and *Foxb1*. Of these, we studied a conserved GABAergic *Glp1r*-expressing population of LepRb neurons (LepRb^Glp1r^) that is concentrated in the DMH. These LepRb^Glp1r^ neurons play major roles in the control of food intake and body weight.

## Results

**Figure 1:**
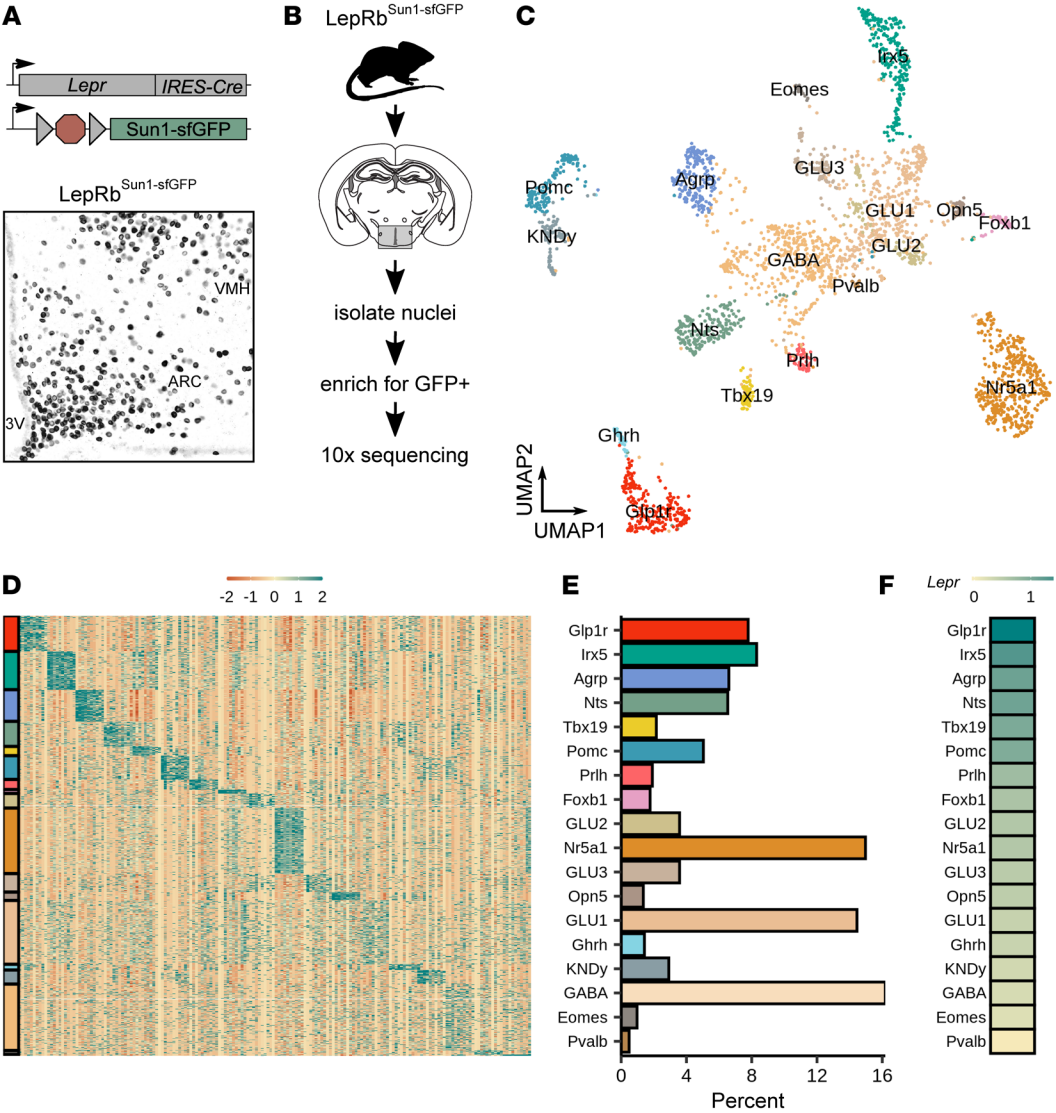
snRNA-Seq of FACS-enriched hypothalamic LepRb neurons defines known and novel LepRb neuron populations. (**A**) Genetic diagram for the LepRb^Sun1-sfGFP^ mouse line (top), and representative image showing GFP immunoreactivity (black) in the mediobasal hypothalamus of a LepRb^Sun1-sfGFP^ mouse (bottom). 3V, third cerebral ventricle. Original magnification, ×10. (**B**) Experimental diagram for isolation of LepRb nuclei from the hypothalamus for snRNA-Seq. (**C**) UMAP projection of all 2,879 hypothalamic LepRb^Sun1-sfGFP^ neuronal nuclei, colored by cluster. (**D**) Scaled expression of top marker genes across all cells; colors on left correspond to colors of populations, as in **C** and **E**. Lowest expression was set to –2; highest expression was set to 2. (**E**) Percentage of cells that map to each cluster. (**F**) Scaled *Lepr* expression across neuron populations within the LepRb-Sun1 data set. Expression in glia was set to 0 and highest Lepr expression was set to 1.

To identify LepRb-expressing hypothalamic cell populations in an unbiased way, we used the LepRb-specific *Lepr^Cre^* allele in combination with a reporter allele that Cre dependently expresses a nuclear-localized Sun1-sfGFP fusion protein (17) in LepRb cells (LepRb^Sun1-sfGFP^, Figure 1A and Supplemental Figure 1; supplemental material available online with this article; https://doi.org/10.1172/JCI157515DS1). We FACS-sorted GFP-containing nuclei from LepRb^Sun1-sfGFP^ hypothalamic tissue and subjected the nuclei to snRNA-Seq using the 10× Genomics 3′ system (Figure 1B). This analysis yielded 4,536 nuclei. We detected more than 600 genes in 4,484 of these nuclei; removing contaminating nonneuronal cell types yielded 3,977 neurons. Following the removal of neurons derived from neighboring nonhypothalamic brain areas (Supplemental Figure 2), we used graph-based clustering of the remaining 2,879 nuclei to identify 18 populations of hypothalamic LepRb neurons (LepRb-Sun1 populations) (Figure 1, C–E). Most of these populations exhibited dozens of unique marker genes (Figure 1D). However, a small number of LepRb-Sun1 populations (e.g., GABA and GLU1) displayed no clear markers and weak transcriptional enrichment; these may represent collections of GFP^–^ contaminants and/or amalgamations of smaller populations of LepRb neurons.

The 18 snRNA-Seq–defined LepRb-Sun1 populations included previously described discrete hypothalamic LepRb cell types, validating our snRNA-Seq approach to identifying populations of hypothalamic LepRb neurons. These previously described populations included those marked by the expression of *Ghrh* (LepRb^Ghrh^ cells, which reside in the ARC); *Nr5a1* (LepRb^Nr5a1^ neurons; ventromedial hypothalamic nucleus [VMH]); *Irx5* (LepRb^Irx5^ neurons; ventral premammillary nucleus); *Agrp* (LepRb^AgRP^ neurons; ARC); *Prlh* (LepRb^Prlh^ neurons; DMH); *Nts* (LepRb^Nts^ neurons; lateral hypothalamic area and DMH); *Pomc* (LepRb^Pomc^ neurons; ARC); and *Kiss1/Tac2/Pdyn* (LepRb^KNDy^ neurons; ARC) (10, 18–24). For all LepRb populations, the marker genes denoted represent those expressed across many neurons of the designated population and displayed expression that was largely restricted to this population; some populations express additional strong marker genes (e.g., *Npy* in LepRb^Agrp^ cells).

We also identified several previously unstudied populations of hypothalamic LepRb neurons with robust gene expression markers, including populations marked by the expression of *Glp1r*, *Tbx19*, *Foxb1*, and *Opn5* (designated as LepRb^Glp1r^, LepRb^Tbx19^, LepRb^Foxb1^, and LepRb^Opn5^ neurons, respectively), all of which exhibited robust gene expression markers. Of all LepRb populations, LepRb^Glp1r^ neurons exhibited the highest level of *Lepr* expression (Figure 1F), suggesting the importance of these cells for leptin action.

To determine whether LepRb^Glp1r^ cells and other novel hypothalamic LepRb populations correspond to specific mediobasal hypothalamic cell types and whether these cell types are conserved in other species, we mapped neurons from our LepRb-Sun1 analysis onto snRNA-Seq–derived cell atlases of the mouse and rat mediobasal hypothalamus that we generated for this purpose and from our previously reported macaque mediobasal hypothalamus snRNA-Seq data set (ref. 25 and Supplemental Figures 3–5). Projecting our LepRb-Sun1 cells into the Uniform Manifold Approximation and Projection (UMAP) embeddings of each data set (Supplemental Figure 4) or coclustering hypothalamic LepRb-Sun1 neurons with neurons from all of these data sets Supplemental Figure 5) revealed many conserved and tightly clustering cell populations that presumably represent bona fide LepRb populations in each species, including LepRb^Glp1r^ neurons, which exhibited high *Lepr* expression across species.

We also mapped translating ribosome affinity purification with RNA-Seq (TRAP-Seq) data from hypothalamic LepRb neurons in aggregate (26) onto the LepRb-Sun1 populations (Supplemental Figure 6); this revealed high expression levels of many leptin-regulated transcripts in several LepRb-Sun1 populations. Mapping an aggregate score for the enrichment of leptin-regulated transcripts onto the LepRb-Sun1 populations revealed that the strongest TRAP-Seq defined transcriptional responses to exogenous leptin are biased toward the LepRb^Glp1r^, LepRb^AgRP^, LepRb^Pomc^, LepRb^Ghrh^, and LepRb^Tbx19^ neuron populations.

**Figure 2:**
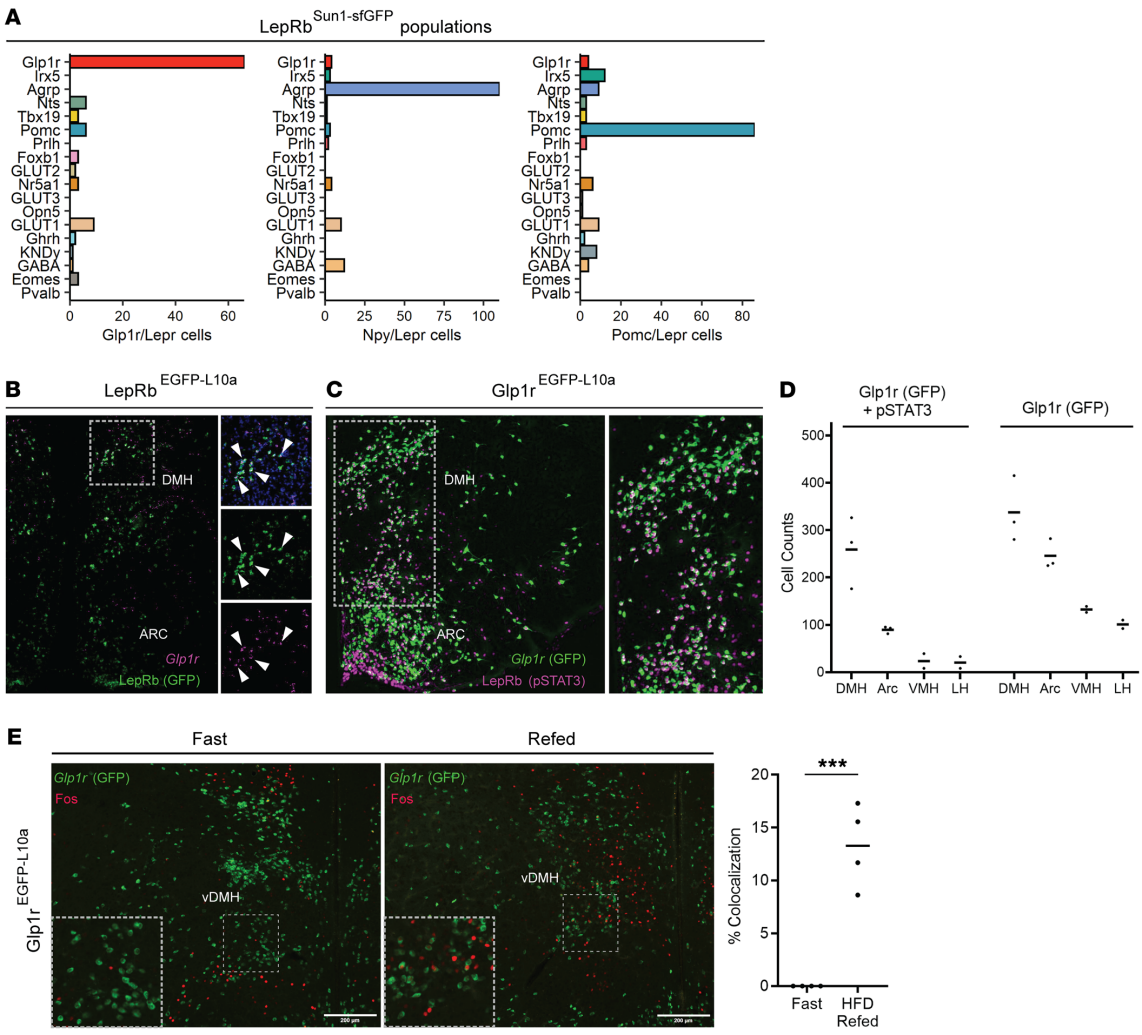
Predominant DMH localization of LepRb^Glp1r^ cells. (**A**) The number of *Glp1r*- (left), *Npy*- (middle), and *Pomc*-expressing (right) cells across LepRb^Sun1-sfGFP^ populations. (**B**) Representative image showing in situ hybridization for *Gfp* [LepRb(GFP), green] and *Glp1r* (magenta) in the hypothalamus of a LepRb^EGFP-L10a^ mouse. Digital zooms of *Glp1r* (bottom), *Gfp* (middle), and merged images (top) are shown. Arrowheads indicate cells demonstrating colocalization. Original magnification, ×4. (**C**) Representative image showing GFP immunoreactivity (-IR) (green) and pSTAT3-IR [LepRb(pSTAT3), magenta] in the hypothalamus of a leptin-treated Glp1r^EGFP-L10a^ mouse. Original magnification, ×4. A digital zoom of the boxed region is shown. (**D**) Quantification of cells containing GFP^+^pSTAT3 or GFP alone across hypothalamic nuclei in mice treated similarly as those shown in **C** (*n* = 3 male animals were assessed). (**E**) Representative images of the DMH showing FOS-IR (red) and *Glp1r* (GFP) in fasted (left; *n* = 3 male and 1 female animal) or fasted and refed with high-fat diet (right; *n* = 2 male and 2 female animals) Glp1r^EGFP-L10a^ mice. Original magnification, ×4. Insets show digital zooms of the boxed regions. Quantification of FOS^+^GFP/GFP neurons is shown in the graph. Scale bar: 200 μm. ***P < 0.001 by Student’s *t* test.

All together, the findings of high *Lepr* expression, robust enrichment of leptin-regulated genes, and conservation across species suggest potentially important roles for the LepRb^Glp1r^ population in leptin action. We thus chose to determine the roles for this LepRb population in leptin action in vivo. As expected, the vast majority of *Glp1r/Lepr*-coexpressing cells from our LepRb-Sun1 analysis mapped to the LepRb^Glp1r^ population (much as *Npy*- and *Pomc*-expressing cells mapped to the LepRb^Agrp^ and LepRb^Pomc^ clusters, respectively) (Figure 2A), suggesting that analysis of *Glp1r*-expressing LepRb cells should reveal the function of the LepRb^Glp1r^ population.

In situ hybridization for *Glp1r* and *Gfp* in LepRb^EGFP-L10a^ mice revealed a substantial population of LepRb^Glp1r^ cells in the DMH (Figure 2B). Using *Glp1r^Cre^* on a reporter strain that Cre-inducibly expresses an EGFP-L10a fusion protein (Glp1r^EGFP-L10a^ mice) similarly revealed the colocalization of leptin-stimulated phosphorylated STAT3 immunoreactivity (pSTAT3-IR, a marker for cell-autonomous leptin action, ref. 27) with GFP in the DMH, with smaller populations of cells in the adjacent ARC and very few cells in the lateral hypothalamic area and VMH (Figure 2, C and D). Few colocalizing neurons were observed outside of the hypothalamus (Supplemental Figure 7). We also directly examined the potential colocalization of POMC-IR with pSTAT3-IR and EGFP in the ARC of leptin-treated Glp1r^EGFP-L10a^ mice, revealing that very few POMC cells contain both *Glp1r^Cre^* and pSTAT3 (Supplemental Figure 7), as previously reported by others (28). Thus, LepRb^Glp1r^ neurons reside predominantly within the DMH, although this population contains some (non-POMC) neurons in the ARC.

Consistent with the notion that leptin poorly mediates the acute regulation of neuronal activity in most cell types, we observed no change in FOS-IR in DMH *Glp1r* neurons following treatment with exogenous leptin (data not shown). However, refeeding following an overnight fast increased FOS-IR in DMH *Glp1r* neurons in a distribution similar to that of LepRb^Glp1r^ neurons (Figure 2E); consistent with the activation of these neurons by signals of nutritional surfeit.

**Figure 3:**
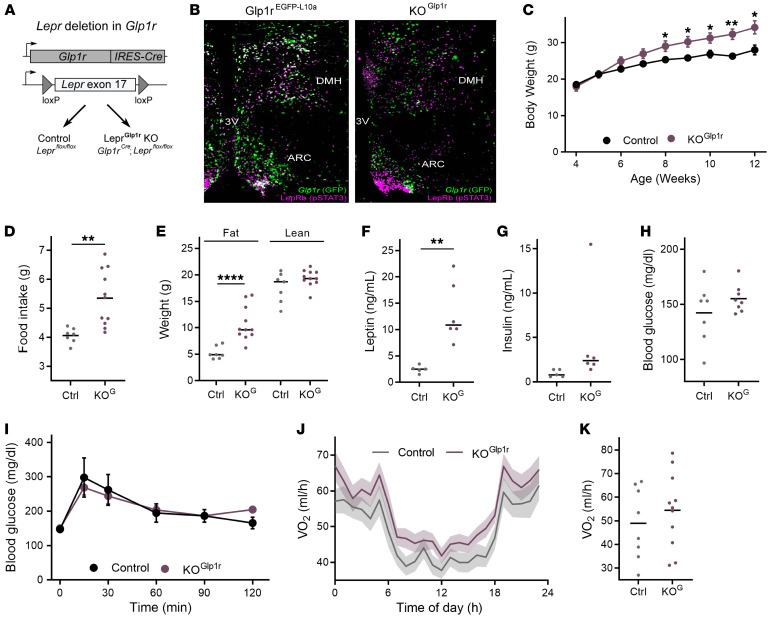
Requirement for *Lepr* in LepRb^Glp1r^ neurons for the control of food intake and body weight by leptin in male mice. (**A**) Experimental design showing the generations of *Glp1r^Cre^;Lepr^fl/fl^* (KO^Glp1r^ or KO^G^) and *Lepr^fl/fl^* control animals. (**B**) Representative images showing leptin-induced pSTAT-IR (LepRb, magenta) and GFP-IR (*Glp1r*, green) in Glp1r^EGFP-L10a^ (left) and KO^Glp1r^ (right) mice. Original magnification, ×4. (**C**–**H**) Body weight (**C**; *n* = 7 Ctrl, *n* = 11 KO^Glp1r^), food intake (**D**; *n* = 7 Ctrl, *n* = 11 KO^Glp1r^), body composition (**E**; *n* = 7 Ctrl, *n* = 11 KO^Glp1r)^, serum leptin (**F**; *n* = 5 Ctrl, *n* = 6 KO^Glp1r)^, serum insulin (**G**; *n* = 5 Ctrl, *n* = 6 KO^Glp1r^), and blood glucose (**H**; *n* = 7 Ctrl, *n* = 11 KO^Glp1r^) in control (black/gray) and KO^Glp1r^ (purple) male mice. (**I**) Glycemic response to an i.p. glucose tolerance test in control (black; *n* = 7) and KO^Glp1r^ (purple; *n* = 11) male mice. (**J** and **K**) VO_2_ measured in metabolic cages across the diurnal cycle (**J**) and averaged over 24 hours (**K**) for KO^Glp1r^ mice (*n* = 11) and control (*n* = 8) male mice. *P < 0.05; **P < 0.01; ***P < 0.001; ****P < 0.0001 by Student’s *t* test.

To determine roles for LepRb^Glp1r^ cells in leptin action, we crossed *Glp1r^Cre^* onto the *Lepr^fl^* background to generate *Glp1r^Cre^;Lepr^fl/fl^* (KO^Glp1r^) mice lacking *Lepr* specifically in *Glp1r*-expressing neurons (Figure 3A). Leptin-stimulated pSTAT3-IR was absent from *Glp1r*-expressing neurons in KO^Glp1r^ neurons, although it was grossly normal otherwise (Figure 3B). Both male (Figure 3, C–E) and female (Supplemental Figure 8) KO^Glp1r^ mice displayed increased body weight characterized by hyperphagia, increased adiposity, and increased circulating leptin concentrations.

While circulating insulin concentrations tended to be increased commensurate with the obesity of these animals (Figure 3F), glucose concentrations remained normal in ad libitum–fed animals and during an i.p. glucose tolerance test (Figure 3, G–I). Furthermore, while LepRb-null mice exhibit severely reduced energy expenditure (29), KO^Glp1r^ mice tended to display increased VO_2_ and other parameters of energy expenditure compared with lean controls, although these trends were not statistically significant (Figure 3, J and K, and Supplemental Figures 8 and 9). The finding that KO^Glp1r^ mice exhibited a similar relationship between VO_2_ and parameters of body weight as did control animals (Supplemental Figures 8 and 9) suggests that leptin action via LepRb^Glp1r^ cells primarily controls food intake (rather than glucose homeostasis or energy expenditure).

We also examined the effect of restoring *Lepr* expression in *Glp1r* cells on an otherwise *Lepr*-null background in *Glp1r^Cre^;Lepr^LSL/LSL^* (Supplemental Figure 10) mice. This maneuver diminished the hyperphagia of *Lepr*-null (KO) mice and improved body weight and adiposity, suggesting the sufficiency of leptin action on LepRb^Glp1r^ neurons for a substantial amount of food intake regulation even in the absence of leptin action on other LepRb populations.

**Figure 4:**
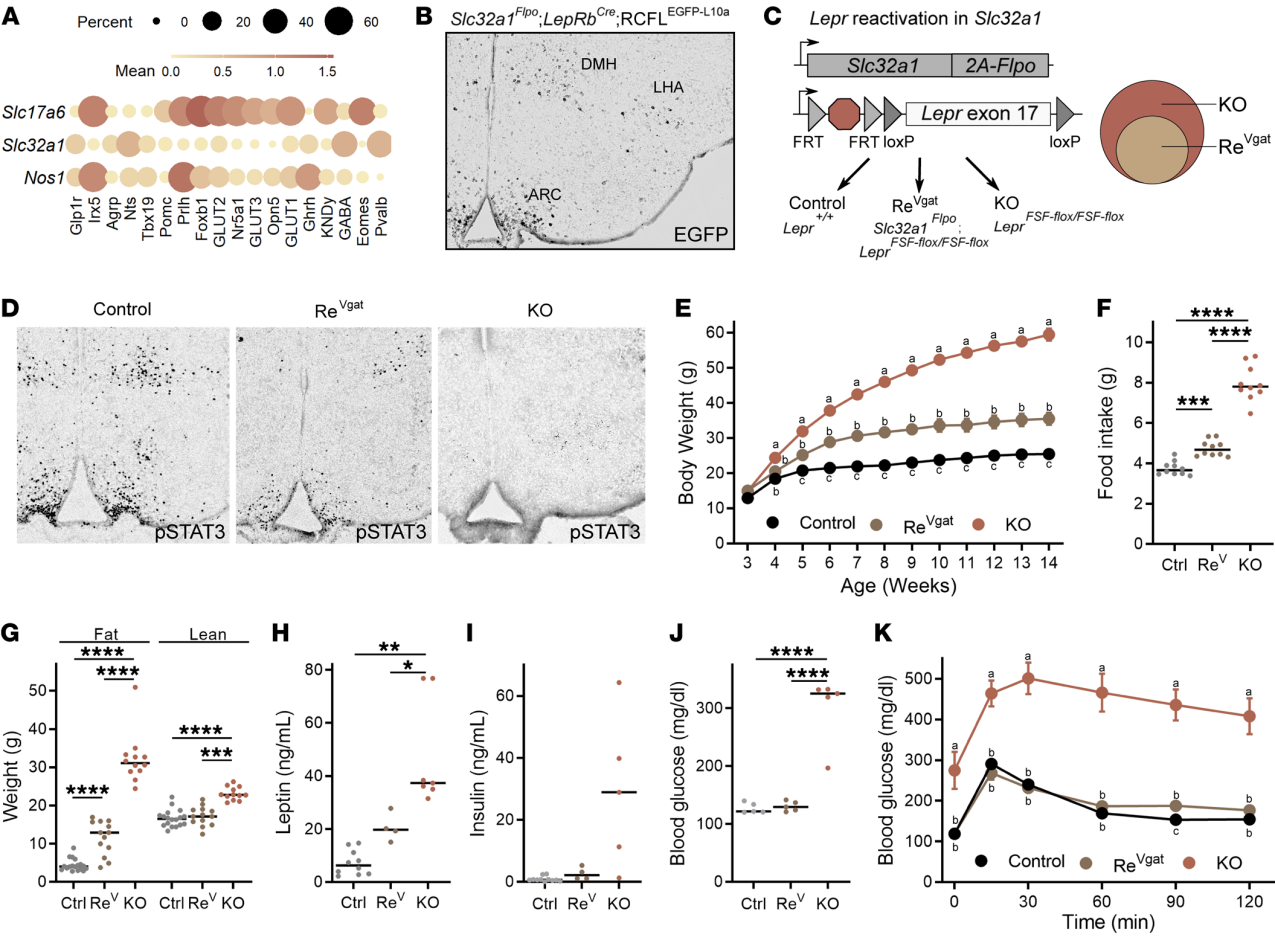
*Lepr* expression in GABAergic Lepr^Glp1r^ cells suffices for most leptin action. (**A**) Expression of *Slc17a6* (vGlut2), *Slc32a1* (vGat), and *Nos1* for LepRb^Sun1-sfGFP^ populations. (**B**) Representative image of GFP-IR (black) in the hypothalami of *Slc32a1^Flpo^*;*LepRb^Cre^* mice on a Flp- and Cre-dependent reporter (RCFL^EGFP-L10a^) background. (**C**) Generation of control, *Lepr^FSF-fl/FSF-fl^* (KO), and *Scl32a1^FlpO^;Lepr^LSL-fl/LSL-fl^* (Re^Vgat^) mice to test the role of *Lepr* in GABAergic neurons for leptin action. (**D**) Representative images showing leptin-stimulated pSTAT3-IR in control, Re^Vgat^, and KO mice. (**E**–**K**) Body weight (**E**; *n* = 11 Ctrl, *n* = 6 Re^Vgat^, *n* = 6 KO male and *n* = 11 Ctrl, *n* = 9 Re^Vgat^, 6 *n* = KO female animals), food intake (**F**; *n* = 11 Ctrl, *n* = 6 Re^V^, *n* = 6 KO male and *n* = 11 Ctrl, *n* = 9 Re^V^, *n* = 6 KO female animals), body composition (**G**; *n* = 10 Ctrl, *n* = 5 Re^V^, *n* = 6 KO male and *n* = 8 Ctrl, *n* = 8 Re^V^, *n* = 6 KO female animals), serum leptin (**H**; *n* = 5 Ctrl, *n* = 2 Re^V^, *n* = 4 KO male and *n* = 5 Ctrl, *n* = 2 Re^V^, *n* = 3 KO female animals), serum insulin (**I**; *n* = 5 Ctrl, *n* = 2 Re^V^, *n* = 2 KO male and *n* = 6 Ctrl, *n* = 3 Re^V^, *n* = 2 KO female animals), blood glucose (**J**; *n* = 11 Ctrl, *n* = 6 Re^V^, *n* = 6 KO male and *n* = 11 Ctrl, *n* = 9 Re^V^, *n* = 6 KO female animals), and glycemic response to i.p. glucose tolerance test (**K**) in control (gray; *n* = 10 male, 9 female), KO (orange; *n* = 4 male, *n* = 5 female animals), and Re^Vgat^ (gold; *n* = 6 male, *n* = 9 female) mice. In **H**–**J**, all mice were ad libitum fed in the AM. Data in **E** and **K** show the mean ± SEM; letters a–c signify conditions that are statistically different (*P* < 0.05) by ANOVA with Tukey’s post hoc test. For **F**–**J**, *P < 0.05; **P < 0.01; ***P < 0.001; ****P < 0.0001 by ANOVA with Dunnett’s post hoc test.

*Lepr* expression in the large sets of GABAergic (marked by the expression of the vesicular GABA transporter [vGAT; encoded by *Slc32a1*]) and *Nos1*-expressing LepRb neurons plays crucial roles in the control of energy balance by leptin (10, 14, 30). Sizable GABAergic LepRb populations include LepRb^Glp1r^, LepRb^Agrp^, LepRb^Ghrh^, and LepRb^Nts^ cells (Figure 4A); of these, LepRb^Glp1r^, LepRb^Ghrh^, and LepRb^Nts^ neurons also exhibited substantial *Nos1* expression. However, the early developmental ablation of *Lepr* from LepRb^Agrp^, LepRb^Ghrh^, or LepRb^Nts^ cells populations only modestly (if at all) affects food intake and energy balance (9, 10, 22). We thus set out to determine whether LepRb^Glp1r^ cells might represent a GABAergic LepRb population crucial for the control of energy balance.

We generated a *Slc32a1^FlpO^* mouse line to permit the selective targeting of GABAergic LepRb populations. Crossing *Slc32a1^FlpO^* mice with *Lepr^Cre^* and a Cre- and Flp-dependent GFP reporter line (31) promoted GFP expression in brain areas known to contain GABAergic, but not glutamatergic (e.g., VMH), LepRb neurons (Figure 4B). To examine the collective ability of GABAergic LepRb neurons to effect the control of energy balance by leptin in the absence of *Lepr* expression in other types of neurons, we crossed *Slc32a1^FlpO^* mice onto the *Lepr^FSF-fl^* mouse line (32), in which an FRT-flanked transcriptional STOP cassette lies upstream of a floxed *Lepr* exon 17, disrupting functional *Lepr* expression (Figure 4C). Thus, the *Lepr^FSF-fl^* allele is null for *Lepr* expression but can be reactivated by Flp (32–34); *Slc32a1^FlpO^;Lepr^FSF-fl/FSF-fl^* (Re^Vgat^) mice are predicted to express *Lepr* only in GABAergic cells.

Indeed, Re^Vgat^ mice exhibited leptin-stimulated pSTAT3-IR in the expected distribution (Figure 4D; like that of GFP in Figure 4B), consistent with restoration of *Lepr* expression in GABAergic cells. These Re^Vgat^ mice displayed dramatic improvements in body weight, food intake, and blood glucose control compared with KO mice (Figure 4, E–K, and Supplemental Figure 11). The remaining difference in energy balance between Re^Vgat^ and control mice did not result from insufficient Flp activity (Flp recombinase activity is notoriously low compared with Cre), because *Slc32a1^Cre^*-mediated *Lepr* reexpression from the Cre-activated *Lepr^LSL^* allele (35) displayed a similar phenotype (Supplemental Figure 12) (36). Hence, the difference in energy balance phenotype between Re^Vgat^ and control mice likely reflects the minor contribution of glutamatergic and other non-GABAergic LepRb cells to the control of energy balance (10, 14).

**Figure 5:**
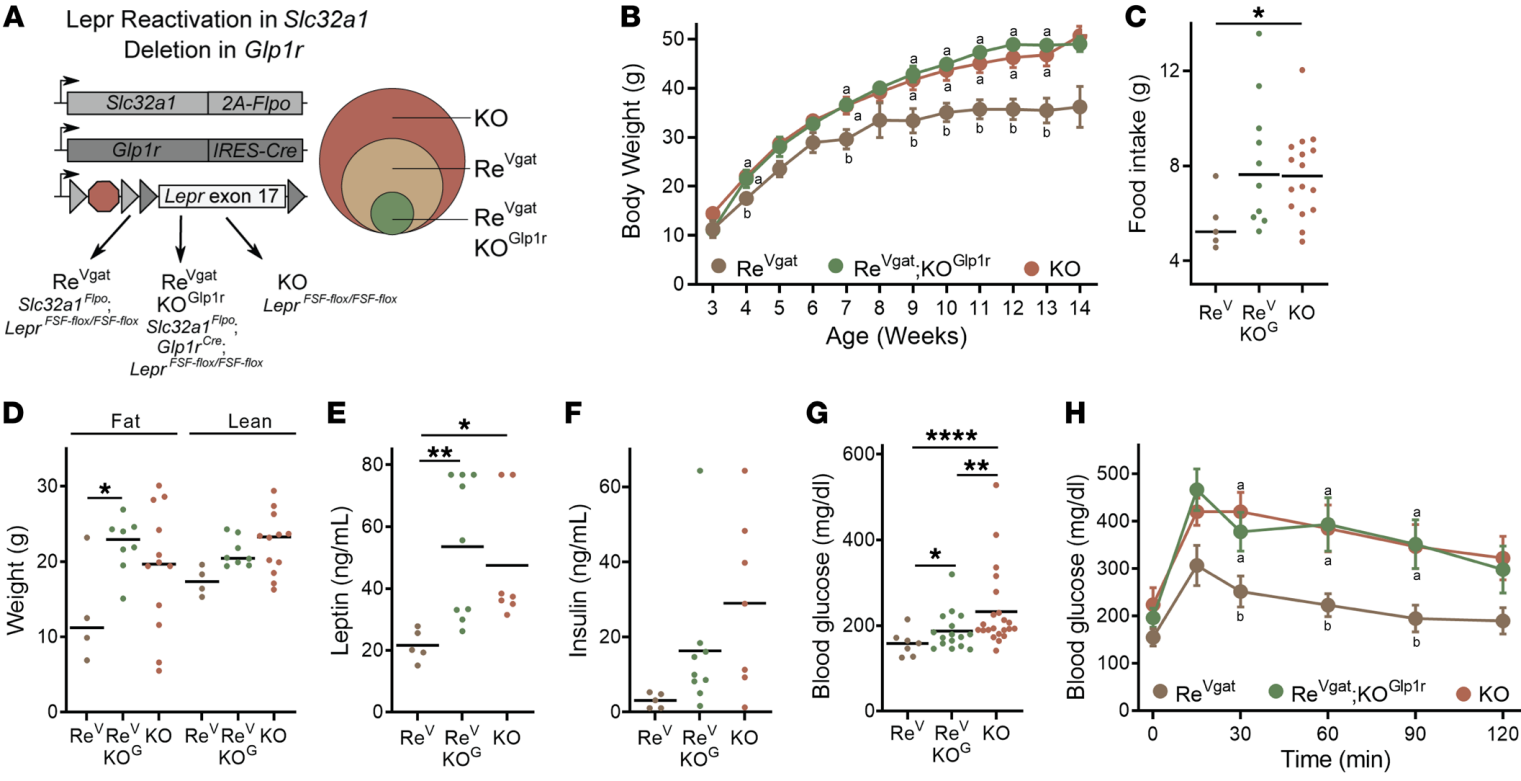
Requirement for *Lepr* in GABAergic *Glp1r* cells for the control of energy balance. (**A**) Generation of *Lepr^FSF-fl/FSF-fl^* (KO), *Scl32a1^FlpO^;Lepr^LSL-fl/LSL-fl^* (Re^Vgat^), and *Scl32a1^FlpO^;Glp1r^Cre^;Lepr^LSL-fl/LSL-fl^* (Re^Vgat^KO^Glp1r^) mice to test the role of *Lepr* in GABAergic *Glp1r* neurons for leptin action. Because of the low number of animals produced by the breeding scheme, data from male and female animals has been combined. (**B**–**H**) Body weight (**B**; *n* = 2 Re^Vgat^, *n* = 6 Re^Vgat^KO^Glp1r^, and *n* = 13 KO male and *n* = 5 Re^Vgat^, *n* = 10 Re^Vgat^KO^Glp1r^, *n* = 9 KO female animals), food intake (**C**; *n* = 2 Re^V^, *n* = 3 Re^V^KO^G^, *n* = 8 KO male and *n* = 3 Re^V^, *n* = 7 Re^V^KO^G^, *n* = 8 KO female animals), body composition (**D**; *n* = 1 Re^V^, *n* = 2 Re^V^KO^G^, *n* = 8 KO male and *n* = 3 Re^V^, *n* = 6 Re^V^KO^G^, *n* = 4 KO female animals), serum leptin and serum insulin (**E** and **F**; *n* = 2 Re^V^, *n* = 2 Re^V^KO^G^, *n* = 4 KO male and *n* = 3 Re^V^, *n* = 7 Re^V^KO^G^, *n* = 3 KO female animals), blood glucose (**G**; *n* = 2 Re^V^, *n* = 6 Re^V^KO^G^, *n* = 13 KO male and *n* = 5 Re^V^, *n* = 10 Re^V^KO^G^, *n* = 9 KO female animals), and glycemic response to i.p. glucose tolerance test (**H**) in Re^vGAT^ (gold; *n* = 1 male and *n* = 5 female), KO (orange; *n* = 8 male and *n* = 5 female), and Re^vGAT^KO^Glp1r^ (green; *n* = 2 male and *n* = 6 female) mice. In **F** and **G**, all mice were ad libitum fed in the AM. Data in **B** and **H** show the mean ± SEM; different letters signify conditions that are statistically different (*P* < 0.05) by ANOVA with Tukey’s post hoc test. For **C**–**H**, *P < 0.05; **P < 0.01; ***P < 0.001; ****P < 0.0001 by ANOVA with Dunnett’s post hoc test.

Because *Lepr* exon 17 is floxed in *Lepr^FSF-fl^* mice, Flp-reactivated *Lepr* expression from this allele can be deactivated by Cre (Figure 5A). Global deletion of the FRT-flanked transcriptional blocker from this previously validated allele forms the basis for the widely used *Lepr^fl^* mouse line (see Figure 3) (8, 10, 18, 32). To determine the contribution of GABAergic LepRb^Glp1r^ cells to the rescue of energy balance control observed in Re^Vgat^ mice, we crossed *Glp1r^Cre^* mice onto the Re^Vgat^ background (where *Lepr* expression is restricted to GABAergic LepRb neurons) to generate *Slc32a1^Flpo^*;*Glp1r^Cre^*;*Lepr^FSF-fl/FSF-fl^* (Re^Vgat^KO^Glp1r^) mice in which *Lepr* expression is restricted to non-*Glp1r* GABAergic neurons (Figure 5A).

Re^Vgat^KO^Glp1r^ mice phenocopied the hyperphagic obesity of KO mice, abrogating the restoration of food intake control, body weight, adiposity, and leptin that was observed in Re^Vgat^ mice (Figure 5, B–E, and Supplemental Figure 13). Although Re^Vgat^KO^Glp1r^ mice exhibited elevated insulin concentrations and impaired glucose tolerance like that of KO mice, their ad libitum–fed blood glucose concentrations were close to normal (Figure 5, F–H, and Supplemental Figure 13), consistent with preserved control of baseline glucose by non-LepRb^Glp1r^ GABAergic *Lepr* cells, despite the obesity-mediated insulin resistance and glucose intolerance of Re^Vgat^KO^Glp1r^ mice. Thus, LepRb^Glp1r^ cells represent a key population of GABAergic LepRb neurons for the control of food intake and energy balance by leptin.

**Figure 6:**
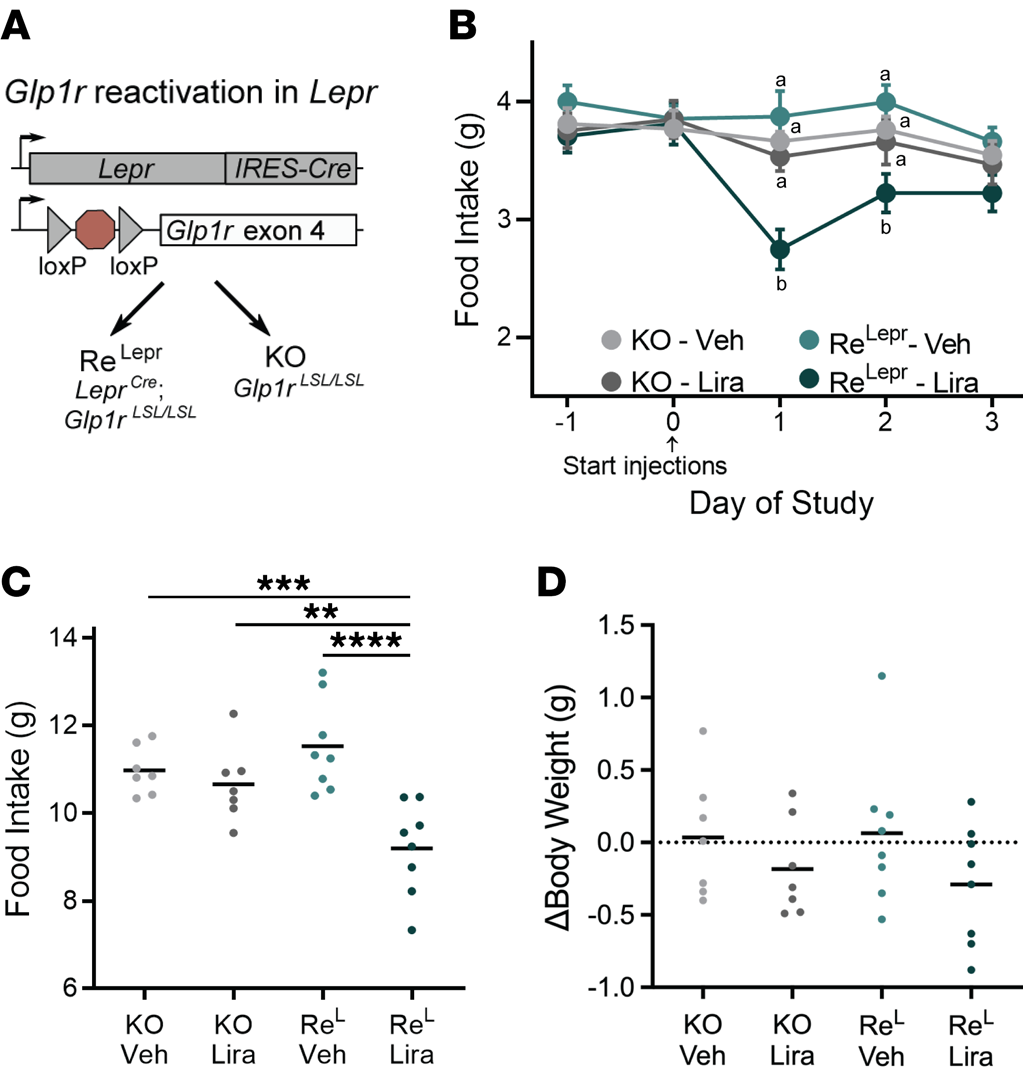
Expression of *Glp1r* in *Lepr* neurons mediates food intake suppression by liraglutide in male mice. (**A**) Generation of *Glp1r^LSL/LSL^* (KO) and *Lepr^Cre^; Glp1r^LSL/LSL^* (Re^Lepr^) mice to test the sufficiency of *Glp1r* expression in *Lepr* cells for food intake suppression by liraglutide. (**B**–**D**) Mice were treated with vehicle for 1 day and then treated with liraglutide (400 μg/d, i.p.) for 3 days (days 0–2) and monitored for food intake and body weight. Daily food intake (**B**), cumulative food intake over the 3-day treatment period (**C**), and change in body weight over the 3-day treatment period (**D**) are shown. *n* = 7 KO animals and 8 Re^L^ animals. Data in shown **B** mean ± SEM; different letters signify conditions that are statistically different (*P* < 0.05) by ANOVA with Tukey’s post hoc test. For **C** and **D**, *P < 0.05; **P < 0.01; ***P < 0.001; ****P < 0.0001 by ANOVA with Tukey’s post hoc test.

Because GLP1R agonists suppress food intake (37) and LepRb^Glp1r^ cells contain *Glp1r*, we examined the potential role for these neurons in the anorectic response to the GLP1R agonist, liraglutide (Figure 6). We crossed *Lepr^Cre^* mice onto the *Glp1r^LSL^* background (in which endogenous *Glp1r* expression is blocked by the presence of a floxed transcription blocker, ref. 38), generating *Lepr^Cre^;Glp1r^LSL/LSL^* (Re^Lepr^) mice that expressed *Glp1r* specifically in LepRb^Glp1r^ neurons for comparison to *Glp1r^LSL/LSL^* (Glp1r-KO) mice (Figure 6A). We subjected these mice to daily injections of vehicle or liraglutide for 3 days, during which time we examined their food intake and body weight. We found that liraglutide treatment reduced food intake for each of the first 2 days of treatment for Re^Lepr^ but not Glp1r-KO mice (Figure 6B), significantly decreasing total food consumed by Re^Lepr^ mice relative to that consumed by Glp1r-KO mice over the 3-day treatment period (Figure 6C). While liraglutide tended to decrease body weight in Re^Lepr^ mice, this effect was not statistically significant (Figure 6D). Thus, GLP1R agonist action on LepRb^Glp1r^ neurons suffices to decrease food intake, but other *Glp1r*-expressing neurons presumably mediate substantial GLP1R agonist–mediated suppression of food intake and body weight.

## Discussion

Our present findings define several conserved populations of hypothalamic LepRb neurons, including LepRb^Glp1r^ neurons that play important roles in the control of food intake by leptin and GLP1R agonists. Additionally, LepRb^Glp1r^ neurons represent a previously postulated population of GABAergic DMH LepRb neurons that plays crucial roles in the control of energy balance (10, 16, 39). The important roles for these neurons in energy balance identify them as potential targets for obesity therapy.

Our data suggest that LepRb^Glp1r^ neurons predominantly control food intake; indeed, ablation of LepRb from these cells increased feeding and adiposity, while energy expenditure was appropriate for body weight in these mice. Furthermore, blood glucose and glucose tolerance remained normal in the KO^Glp1r^ mice despite their adiposity, and blood glucose remained lower than KO mice in Re^Vgat^KO^Glp1r^ mice, despite their equivalent body weight and adiposity. These findings suggest that LepRb^Glp1r^ neurons play little role in the control of blood glucose other than effects secondary to the control of adiposity and that the LepRb neurons that control feeding differ somewhat from those that directly control glucose homeostasis. Hence, the dysregulation of feeding and glucose control in obesity and type 2 diabetes, respectively, might result from insults to separate circuits.

We also speculate that KO^Glp1r^ mice retain normal parameters of other endocrine functions. Indeed, not only do KO^Glp1r^ mice exhibit normal energy expenditure (suggesting normal sympathetic tone and thyroid function), but also we routinely breed KO^Glp1r^ mice (data not shown), which is not possible with KO mice (40).

Not only is the obesity phenotype of KO^Glp1r^ mice more prominent than the reported phenotypes for mice lacking *Lepr* in *Pomc* or *Agrp* neurons since early development (9, 10, 41), but KO^Glp1r^ mice do not exhibit the alterations in glucose homeostasis and/or energy expenditure displayed by these other models. Interestingly, in contrast to the mild phenotype observed following the early developmental deletion of *Lepr* from *Agrp* neurons, ablating *Lepr* from *Agrp* neurons in adults promotes dramatic hyperphagia and obesity (9–11), consistent with developmental compensation for early perturbations to *Agrp* neurons (12) and suggesting that the hyperphagic obesity of *Lepr*-null mice must result from the loss of *Lepr* in non-*Agrp* neurons (such as LepRb^Glp1r^ cells).

Previous authors have studied roles for *Lepr* in anatomically (rather than genetically) defined DMH neurons. While LepRb^Glp1r^ neurons presumably overlap extensively with ventral DMH *Lepr* neurons previously shown to modulate feeding (16), LepRb^Glp1r^ cells likely overlap little with the dorsal DMH *Lepr* neurons that control energy expenditure (15). The dorsal DMH *Lepr* neuron population likely overlaps with LepRb^Prlh^ neurons previously shown to control energy expenditure (21). However, other populations of DMH *Lepr* neurons might also contribute.

Like ARC *Pomc* and *Agrp* neurons, LepRb^Glp1r^ neurons presumably respond to stimuli other than leptin. Indeed, our finding that refeeding promotes the accumulation of FOS-IR in DMH *Glp1r* neurons distributed similarly to LepRb^Glp1r^ neurons suggests the potential for their acute activation by food intake. Furthermore, peripherally administered GLP1R agonists accumulate in the DMH (42) and can drive significant weight loss in humans (37), consistent with the potential modulation of LepRb^Glp1r^ neurons by exogenous GLP1R agonists and/or endogenous GLP-1. Indeed, in addition to mediating the control of food intake and body weight in response to endogenous leptin signaling, LepRb^Glp1r^ neurons can mediate GLP1R agonist–stimulated suppression of food intake, since reactivation of *Glp1r* expression in these cells in an otherwise *Glp1r*-null background enables the suppression of food intake by liraglutide. However, *Glp1r* expression in LepRb^Glp1r^ neurons mediates little control of body weight by liraglutide, suggesting important roles for other *Glp1r* neurons in the major food intake and weight loss effects of liraglutide. Indeed, previous work has shown that *Glp1r* in glutamatergic (rather than GABAergic) neurons mediates the dominant effects of GLP1R agonists on these parameters (38).

While the downstream neural targets of LepRb^Glp1r^ neurons remain unknown, tracing projections from *Lepr*- or *Glp1r*-expressing neurons in the DMH revealed that these cells predominantly innervate the ARC and PVH (Supplemental Figure 14). Together with the observation that LepRb^Glp1r^ neurons primarily control food intake (rather than energy expenditure or glucose homeostasis) in response to endogenous leptin action, this projection pattern suggests that LepRb^Glp1r^ neurons might modulate the activity of ARC *Agrp* or PVH *Mc4r* cells. While it remains possible that LepRb^Glp1r^ neurons could modulate the activity of ARC *Pomc* cells, *Pomc* cells play more prominent roles in the modulation of blood glucose and energy expenditure than ARC *Agrp* or PVH *Mc4r* cells (35, 41, 43), which primarily modulate food intake like LepRb^Glp1r^ cells.

Furthermore, the finding that refeeding activates a population of DMH *Glp1r* neurons that lie in a distribution similar to that of LepRb^Glp1r^ neurons is consistent with a model in which GABAergic LepRb^Glp1r^ neurons innervate and inhibit ARC *Agrp* neurons in response to signals of nutritional surfeit, including those from the gut. Indeed, earlier findings suggest that a previously unidentified population of feeding activated GABAergic DMH LepRb neurons project to and inhibit *Agrp* neurons to control food intake (39). In future work, it will be interesting to examine the possibility that LepRb^Glp1r^ neurons serve this function.

Our snRNA-Seq analysis also identified several additional potentially novel populations of hypothalamic LepRb neurons, including LepRb^Tbx19^, LepRb^Foxb1^, and LepRb^Opn5^ cells, all of which express substantial *Lepr* and some of which exhibit clear cross-species conservation. In future studies it will be important to define the roles and mechanisms of action for these LepRb populations in leptin action. Similarly, analysis of more LepRb neurons from LepRb^Sun1-sfGFP^ mice may reveal additional conserved populations from within the GABA and GLU1-3 populations that currently exhibit poor differentiation; it will be important to determine physiologic roles for these populations as they are more clearly defined.

## Methods

### Animals.

Mice and rats were bred in the Unit for Laboratory Animal Medicine at the University of Michigan. Animals were provided with ad libitum access to food (Purina Lab Diet 5001) and water in temperature-controlled (25°C) rooms on a 12-hour light/dark cycle with daily health status checks.

The *Glp1r^Cre^* mouse line (The Jackson Laboratory, 029283) was a gift from Stephen Liberles (Harvard Medical School, Boston, Massachusetts, USA) (44). *Lepr^fl^* and *Lepr^FSF-fl^* lines were gifts from Streamson Chua (Albert Einstein College of Medicine, New York, New York, USA) (32). The *Lepr^loxTB^* mouse line (The Jackson Laboratory, 018989) was a gift from Joel Elmquist (University of Texas Southwestern, Dallas, Texas, USA) (35). *ROSA26 ^CAG-LSL-eGFP-L10a^* (ROSA26^EGFP-L10a^) mice (26), *Lepr^Cre^* mice (The Jackson Laboratory, 032457) (26), *ROSA26^CAG-LSL-FSF-eGFP-L10a^* (RCFL^EGFP-L10a^) (31), *ROSA26^CAG-Sun1/sfGFP^* (The Jackson Laboratory, 030952) (17), Slc32a1Cre (The Jackson Laboratory, 028862) (14), and *Glp1r^LSL^* mice (38) have been described previously. *Slc32a1^Flpo^* mice were generated for this study (see below for details).

### Generation of the Slc32a1^Flpo^ mouse line.

Slc32a1-2A-Flpo (*Slc32a1^Flpo^*) mice were generated using recombineering techniques as previously described (45). Briefly, the Flpo transgene (AddGene plasmid 13793) and a loxP-flanked neomycin selection cassette were subcloned after 2A self-cleaving peptide. The 2A-Flpo-neomycin cassette was then targeted 3 bp downstream of the stop codon of *Slc32a1* in a bacterial artificial chromosome. The final targeting construct containing the *Slc32a1*-*2A-Flpo* neomycin cassette and 4 kb of flanking genomic sequence on both sides was electroporated into ES cells followed by neomycin selection. Appropriately targeted clones were identified by quantitative PCR and confirmed by Southern blot analysis. Targeted clones were expanded and injected into blastocysts at the University of Michigan Transgenic Core. Chimeric offspring were then bred to confirm germline transmission of the *Slc32a1*-*2A-Flpo* allele; the neomycin selection cassette was removed by breeding to the E2A-Cre deleter strain (The Jackson Laboratory, 003724).

### Generation of study animals.

*Lepr^Cre/Cre^* animals were crossed to *ROSA26^CAG-Sun1/sfGFP/CAG-Sun1/sfGFP^* mice to generate *Lepr^Cre/+^;ROSA26^CAG-Sun1/sfGFP/+^* (LepRb^Sun1-sfGFP^) mice for study. We generated *Glp1r^Cre/+^;Lepr^fl/fl^* and *Lepr^fl/fl^* animals by intercrossing *Glp1r^Cre/+^;Lepr^fl/fl^* and *Lepr^fl/fl^* mice. We generated mice with reactivation of *Lepr* in vGat neurons (and control mice) by crossing *Slc32a1^Flpo/+^;Lepr^FSF-fl/+^* mice with *Lepr^FSF-fl/+^* animals (Flp-dependent reactivation) or *Slc32a1^Cre/+^;Lepr^loxTB/+^* mice with *Lepr^loxTB/+^* animals (Cre-dependent reactivation). To generate animals with reactivation of *Lepr* in Vgat neurons and *Lepr* deactivation in *Glp1r* neurons (and their controls), we crossed *Slc32a1^Flpo/+^;Lepr^FSF-fl/+^* mice with Glp1r^Cre;^*Lepr^FSF-fl/+^* animals. To generate mice with reactivation of *Glp1r* in *Lepr* neurons, we intercrossed *Lepr^Cre/Cre^;Glp1r^LSL/LSL^* animals; controls were derived by intercrossing *Glp1r^LSL/LSL^* mice.

### Longitudinal study.

Mice were maintained on standard chow diet and body weight was measured weekly from 3 to 4 weeks of age. Blood glucose was measured biweekly from 5 weeks of age. Some cohorts were single housed to measure food intake weekly; other cohorts were single housed from 12 to 14 weeks of age for food intake measurement. At 15 weeks of age, mice were subjected to a glucose tolerance test. Following a 4- to 5-hour fast, mice were injected with D-glucose (2 g/kg, i.p.), and blood glucose was measured at 0-, 15-, 30-, 60-, 90-, and 120-minute time points. Mice were subjected to body composition measurements (Bruker, Minispec LF 90II) at 17 weeks. Blood for the determination of serum leptin and insulin concentrations was taken at the time of euthanasia at 18 weeks. Blood for glucose determinations was collected from the tail vein and measured using a OneTouch Ultra 2 glucometer (Johnson & Johnson).

### Tissue prep, cDNA amplification, and library construction for 10× snRNA-Seq.

Mice and rats were euthanized using isoflurane and decapitated; then, the brain was subsequently removed from the skull and sectioned into 1 mm thick coronal slices using a brain matrix. The mediobasal hypothalamus was dissected and flash frozen in liquid N_2_.

On the day of the experiment, frozen tissue (from 2 to 3 mixed sex mice or 1 rat per sample) was homogenized in Lysis Buffer (EZ Prep Nuclei Kit, Sigma-Aldrich) with Protector RNAase Inhibitor (Sigma-Aldrich) and filtered through a 30 mm MACS strainer (Miltenyi). Strained samples were centrifuged at 500 rcf for 5 minutes at 4°C, and pelleted nuclei were resuspended in wash buffer (10 mM Tris Buffer, pH 8.0, 5 mM KCl, 12.5 mM MgCl_2,_ 1% BSA with RNAse inhibitor). Nuclei were strained again and recentrifuged at 500 rcf for 5 minutes at 4°C. Washed nuclei were resuspended in wash buffer with propidium iodide (Sigma-Aldrich), and stained nuclei underwent FACS sorting on a MoFlo Astrios Cell Sorter. For LepRb-Sun1 experiments, GFP^+^ and PI^+^ nuclei were collected. For all other experiments, only PI^+^ nuclei were collected; we ran 1 mixed-sex mouse sample (1 library) and 2 rat samples (1 of each sex; 1 library each). Sorted nuclei were centrifuged at 100 rcf for 5 minutes at 4°C and resuspended in wash buffer to obtain a concentration of 750–1,200 nuclei/μL. RT mix was added to target approximately 10,000 nuclei recovered and loaded onto the 10× Chromium Controller chip. The Chromium Single Cell 3′ Library and Gel Bead Kit v3, Chromium Chip B Single Cell kit, and Chromium i7 Multiplex Kit were used for subsequent RT, cDNA amplification, and library preparation, as instructed by the manufacturer. Libraries were sequenced on an Illumina NovaSeq 6000 (pair ended with read lengths of 150 nt).

### snRNA-Seq data analysis.

FASTQ files were mapped to the appropriate genome (Ensembl GRCm38 or Rnor_6.0) using cellranger to generate count matrix files, and data were analyzed in R. Macaque count matrix files were downloaded from GEO data set GSE172203. Genes expressed in at least 5 cells in were retained. Cells with at least 600 detected genes were retained. Doublets were scored using Scrublet (46); clusters with a median doublet score of greater than 0.3 or individual cells with scores greater than 0.3 were removed.

The data were then normalized using scran (47) and centered and scaled for each data set independently, and genes that were called variable by Seurat *FindVariableFeatures* were input to PCA. The top PCs were retained at the “elbow” of the scree plot (normally 15–30, depending on the data set) and then used for dimension reduction using UMAP and clustering using the Seurat *FindNeighbors* and *FindClusters* functions. *FindClusters* was optimized for maximizing cluster consistency by varying the resolution parameter from 0.2 upward in steps of 0.2 until a maximal mean silhouette score was found. Clusters were then hierarchically ordered based on their Euclidean distance in PC space and ordered based on their position in the tree.

Cell types were identified by projecting labels from a published hypothalamic single-cell RNA-Seq data set (GSE87544) using the Seurat CCA method. Cells were labeled by the highest scoring cell type for the cell and its 14 nearest neighbors (from Seurat SNN). Neuron cluster names were chosen based on genes found in unbiased marker gene search (Seurat *FindMarkers*). Clusters without unique marker genes were labeled by their neurochemical identity (GABA or GLU).

### TRAP-Seq analysis.

Published TRAP-Seq data (GSE162603) were used to identify signatures of hypothalamic Lepr cells. Enriched genes were determined using DESeq2, including an effect of sample pair in the model to account for pairing of the bead–sup samples (~ Pair + Cells). Effects of leptin were identified using only bead samples with 10-hour treatment (PBS or leptin) and a DESeq2 model of ~genotype (WT or *ob*/*ob*) + treatment.

Association of leptin-regulated genes and LepRb^Sun1-sfGFP^ populations was performed by projecting the scaled expression of significant leptin-regulated genes into principal component space. The association score is the magnitude of the first principal component.

### Species integration.

Mouse (approximately 6,300 whole hypothalamic neurons that passed quality control), rat (approximately 16,500 whole hypothalamic neurons that passed quality control), and macaque data sets were processed in the same way as for LepRb^Sun1-sfGFP^, including identifying cell types and neuron clusters. For rat and macaque, the data were first converted into 1:1 orthologs using the mouse Ensembl annotation from biomaRt. To project LepRb^Sun1-sfGFP^ cells onto the UMAP embeddings of each species, we used Seurat’s *MapQuery* function with the CCA reduction. To identify ortholog populations, we generated a combined data set for all species using only their ortholog genes. Data sets were harmonized using Harmony and then clustered as previously. Clusters containing 80% of cells from each LepRb^Sun1-sfGFP^ population were labeled based on the LepRb^Sun1-sfGFP^ name. If more than one LepRb^Sun1-sfGFP^ population had 80% of its cells associated with the conserved cluster, these clusters were subclustered and the subcluster containing the most LepRb^Sun1-sfGFP^ cells from each cluster was assigned that name. Leptin-response scores were generated using the same approach as for the LepRb^Sun1-sfGFP^ data set.

### Immunostaining.

Mice were euthanized with isoflurane and then perfused with PBS for 5 minutes followed by an additional 5 minutes of 10% formalin. For immunohistochemistry of pSTAT3, mice were injected with leptin (5 mg/kg) 1 hour before euthanasia. Brains were then removed and post-fixed in 10% formalin for 4 hours at room temperature, before being moved to 30% sucrose until sunk. Brains were then sectioned as 30 mm thick free-floating sections and stained. Sections were treated sequentially with 1% hydrogen peroxide/0.5% sodium hydroxide, 0.3% glycine, 0.03% sodium dodecyl sulfate, and blocking solution (PBS with 0.1% triton, 3% normal donkey serum; Thermo Fisher Scientific).

### Immunohistochemical and immunofluorescent staining.

The sections were incubated overnight at room temperature using standard procedures for the primary and secondary antibodies below. The following day, sections were washed and incubated with either biotinylated antibodies (1:200, Jackson Immunoresearch, 711-065-152) followed by avidin-biotin complex amplification and 3,3-diaminobenzidine (Thermo Fisher Scientific, 32020 and 34065) or fluorescent secondary antibodies with species-specific Alexa Fluor 488 or 568 (1:250, Invitrogen, A-11039 or A-11011) to visualize proteins. Primary antibodies used include GFP (1:1,000, Aves Laboratories, 1020), FOS (1:1,000, Cell Signaling, 2250), dsRed (1:1,000, Takara Bio, 632496), POMC (1:1,000, Phoenix Pharmaceuticals Inc., H-029-30), and pSTAT3 (1:500, Cell Signaling, 9145). Images were collected on an Olympus BX51 or Olympus BX53 microscope. Images were background subtracted and enhanced by shrinking the range of brightness and contrast in ImageJ (NIH).

### RNAscope.

In situ hybridization was performed with the RNAscope Multiplex Fluorescent Assay v2 from Advanced Cell Diagnostics (ACD) combined with Tyramide Signal Amplification technology (TSA) and dyes from Akoya Biosciences. Fresh frozen brains collected from 10-week-old Lepr^eGFP-L10a^ female mice were sliced on a cryostat at 16 μm and mounted directly onto Superfrost Plus slides (Fisher Scientific). The tissue was fixed in 10% neutral buffered formalin for 15 minutes and then dehydrated in ethanol. Endogenous peroxidase was blocked with H_2_O_2_ for 10 minutes, washed in DEPC water, and then tissue was gently digested for 30 minutes at room temperature using Protease IV from the ACD kit. The tissue was incubated for 2 hours at 40°C using probes targeting EGFP, Mm-Ebf1, Mm-Glp1r (418851 ACD), and ACD’s 3-plex RNAscope Positive Control Probes for mouse tissue. After hybridization, probes were labeled with Akoya dyes using TSA technology. Sections were counterstained with DAPI and cover slipped with ProLong Gold antifade mounting medium (Thermo Fisher Scientific).

### Statistics.

All plotting and statistical analysis were performed using R 4.1.1. Specific statistical tests are listed in the figure legends and include 2-tailed Student’s *t* test, Tukey’s post hoc test, 2-way ANOVA with Dunnett’s post hoc test, and 2-way ANOVA with Tukey’s post hoc test. Sample sizes and sex distribution are listed in Supplemental Table 1.

### Study approval.

All procedures performed were approved by the University of Michigan Committee on the Use and Care of Animals and were in accordance with the Association for Assessment and Accreditation of Laboratory Animal Care and NIH guidelines.

### Data availability.

Requests for further information and for resources and reagents should be directed to and will be fulfilled by MGM. All materials related to this study, including *Slc32a1^Flpo^* mice produced for this study, are available upon reasonable request. Sequencing data, count matrix files, and metadata are available through the Gene Expression Omnibus database (GEO GSE172463). Analysis scripts for all sequencing results presented in the paper are available at https://github.com/ajtomlinson59/-rupp_tomlinson-jclininvest-2023 (commit: 0e0569491f78809d2ca149dce592936eb6bb840c). All other data (i.e., mouse phenotyping data) are available from MGM upon request. Mouse models will be made available upon reasonable request. Reagents will be made freely available to academic laboratories.

## Author contributions

ACR, AJT, CL, LBK, RJS, DPO, PK, and MGM conceptualized the study. ACR, AJT, AHA, PK, JPW, and MGM provided study methodology. ACR provided informatic analysis. ACR, AJT, AHA, WTY, AMD, CT, SRL, MAK, and AM provided investigation. DPO provided resources. ACR curated data. ACR and MGM wrote the original draft of the manuscript. All authors reviewed and edited the manuscript. ACR, AJT, and MGM provided visualization. DPO, CL, and LBK provided supervision. PK and MGM acquired funding.

## Supplementary Material

Supplemental data

Supporting data values

## Version 1 08/15/2023

In-Press Preview

## Version 2 10/02/2023

Electronic publication
