# Supplementary material for: Suppression of food intake by Glp1r/Lepr-coexpressing neurons prevents obesity in mouse models
Source: J Clin Invest. 2023 Oct 2;133(19):e157515. doi: 10.1172/JCI157515 (PMC10541203; doi:10.1172/JCI157515)
Supplement: Supplemental data [file jci-133-157515-s140.pdf]

Supplemental Tables and Figures

Supplemental Table 1. Sample size and sex distribution for figures.

			N (Males)	N (Females)
Figure 2	D	Pstat3	3	-
	F	Fast	3	1
		Refed	2	2
Figure 3	C, D, E, H, I	Ctrl	7	-
		Lepr^{Glp1R}KO	11	-
	F, G	Ctrl	5	-
		Lepr^{Glp1R}KO	6	-
	J, K	Ctrl	8	-
		Lepr^{Glp1R}KO	11	-
Figure 4	E, F, J	Ctrl	11	11
		Re^{Vgat}	6	9
		KO	6	6
	G	Ctrl	10	8
		Re^{Vgat}	5	8
		KO	6	6
	H	Ctrl	5	5
		Re^{Vgat}	2	2
		KO	4	3
	I	Ctrl	5	6
		Re^{Vgat}	2	2
		KO	2	3
	K	Ctrl	10	9
		Re^{Vgat}	6	9
		KO	4	5
Figure 5	B, G	Re^{Vgat}	2	5
		Re^{Vgat}KO^{Glp1R}	6	10
		KO	13	9
	C	Re^{Vgat}	2	3
		Re^{Vgat}KO^{Glp1R}	3	7
		KO	8	8
	D	Re^{Vgat}	1	3
		Re^{Vgat}KO^{Glp1R}	2	6
		KO	8	3
	E, F	Re^{Vgat}	2	3
		Re^{Vgat}KO^{Glp1R}	2	7
		KO	4	3
	H	Re^{Vgat}	1	5
		Re^{Vgat}KO^{Glp1R}	2	6
		KO	8	5
Figure 6	A-D	KO – Veh	7	-
		KO – Lira	7	-

		Re^{L} – Veh	8	-
		Re^{L} – Lira	8	-
S8	A-L	Ctrl	8	-
		KO	11	-
	M-P	Ctrl	8	-
		KO	10	-
S9	A, B, F	Ctrl	-	10
		KO	-	9
	C	Ctrl	-	15
		KO	-	11
	D, E	Ctrl	-	6
		KO	-	6
	G-R	Ctrl	-	7
		KO	-	8
	S-V	Ctrl	-	7
		KO	-	7
S10	B, E	Ctrl	11	5
		Re^{Glp1R}	4	5
		KO	12	7
	C	Ctrl	9	4
		Re^{Glp1R}	3	4
		KO	11	6
	D	Ctrl	7	3
		Re^{Glp1R}	2	4
		KO	5	5
	F	Ctrl	9	1
		Re^{Glp1R}	2	4
		KO	8	5
S11	A, B, F	Ctrl	11	-
		Re^{Vgat}	6	-
		KO	6	-
	C	Ctrl	10	-
		Re^{Vgat}	5	-
		KO	6	-
	D	Ctrl	5	-
		Re^{Vgat}	2	-
		KO	4	-
	E	Ctrl	5	-
		Re^{Vgat}	2	-
		KO	2	-
	G	Ctrl	10	-
		Re^{Vgat}	6	-
		KO	4	-
	H, I, M	Ctrl	-	11
		Re^{Vgat}	-	9
		KO	-	6
	J	Ctrl	-	8
		Re^{Vgat}	-	8
		KO	-	6

	K	Ctrl	-	5
		Re^{Vgat}	-	2
		KO	-	3
	L	Ctrl	-	6
		Re^{Vgat}	-	2
		KO	-	3
	N	Ctrl	-	9
		Re^{Vgat}	-	9
		KO	-	5
S12	A-D	Ctrl	10	-
		Re^{Vgat}	7	-
		KO	3	-
	E-H	Ctrl	-	8
		Re^{Vgat}	-	4
		KO	-	6
S13	A, F	Re^{Vgat}	2	-
		$Re^{Vgat}KO^{Glp1R}$	6	-
		KO	13	-
	B	Re^{Vgat}	2	-
		$Re^{Vgat}KO^{Glp1R}$	3	-
		KO	8	-
	C	Re^{Vgat}	1	-
		$Re^{Vgat}KO^{Glp1R}$	2	-
		KO	8	-
	D, E	Re^{Vgat}	2	-
		$Re^{Vgat}KO^{Glp1R}$	2	-
		KO	4	-
	G	Re^{Vgat}	1	-
		$Re^{Vgat}KO^{Glp1R}$	2	-
		KO	8	-
	H, M	Re^{Vgat}	-	5
		$Re^{Vgat}KO^{Glp1R}$	-	10
		KO	-	9
	I	Re^{Vgat}	-	3
		$Re^{Vgat}KO^{Glp1R}$	-	7
		KO	-	8
	J	Re^{Vgat}	-	3
		$Re^{Vgat}KO^{Glp1R}$	-	6
		KO	-	3
	K, L	Re^{Vgat}	-	3
		$Re^{Vgat}KO^{Glp1R}$	-	7
		KO	-	3
	N	Re^{Vgat}	-	5
		$Re^{Vgat}KO^{Glp1R}$	-	6
		KO	-	5

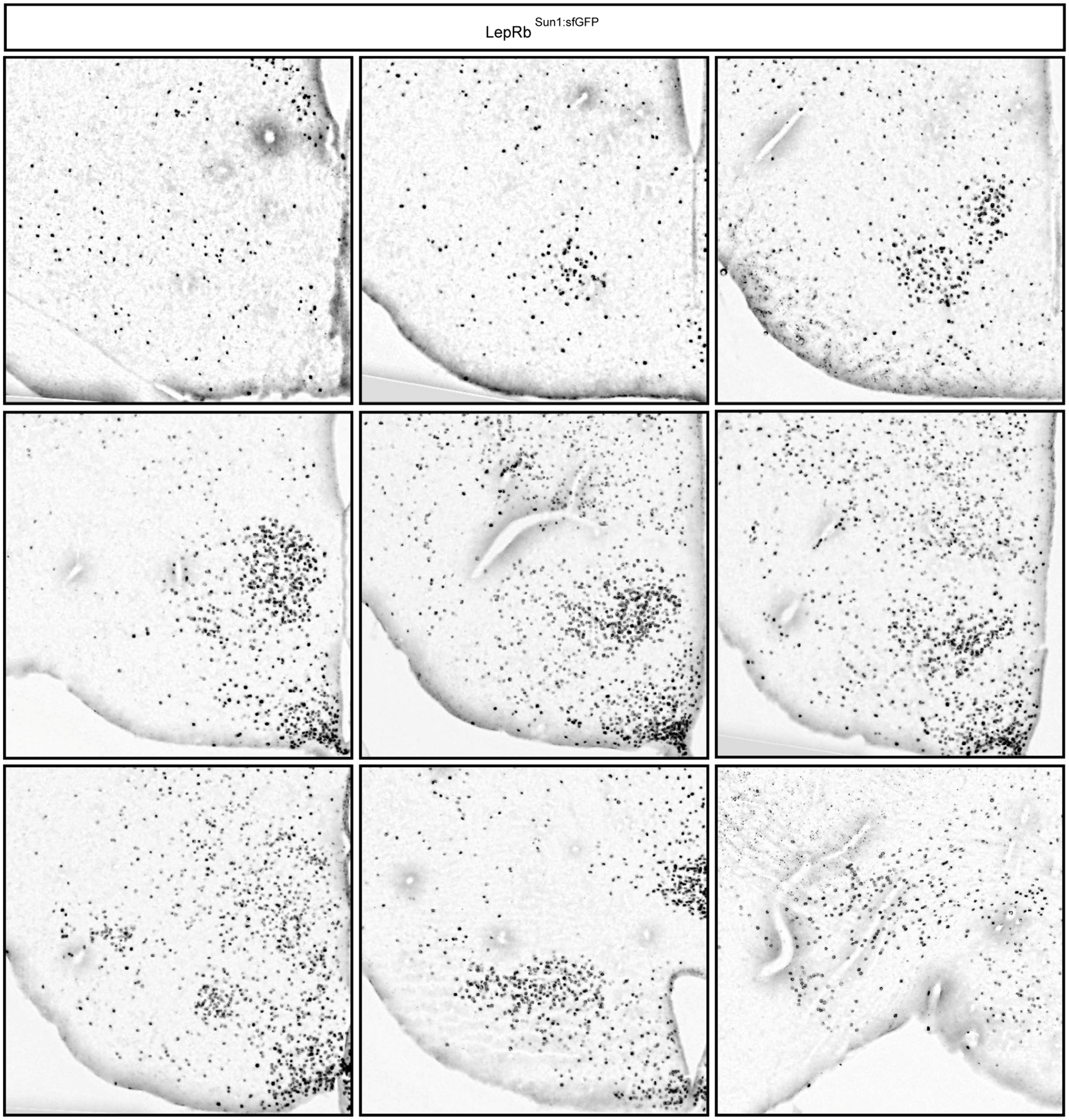

Supplemental Figure 1. SUN1-sfGFP detection the hypothalamus of LepRb$^{Sun1\text{-}sfGFP}$ mice. Representative images of GFP-IR (black, signal inverted) in the hypothalamus of a LepRb$^{Sun1\text{-}sfGFP}$ mouse. Images are shown rostral to caudal going left to right and then down the rows.

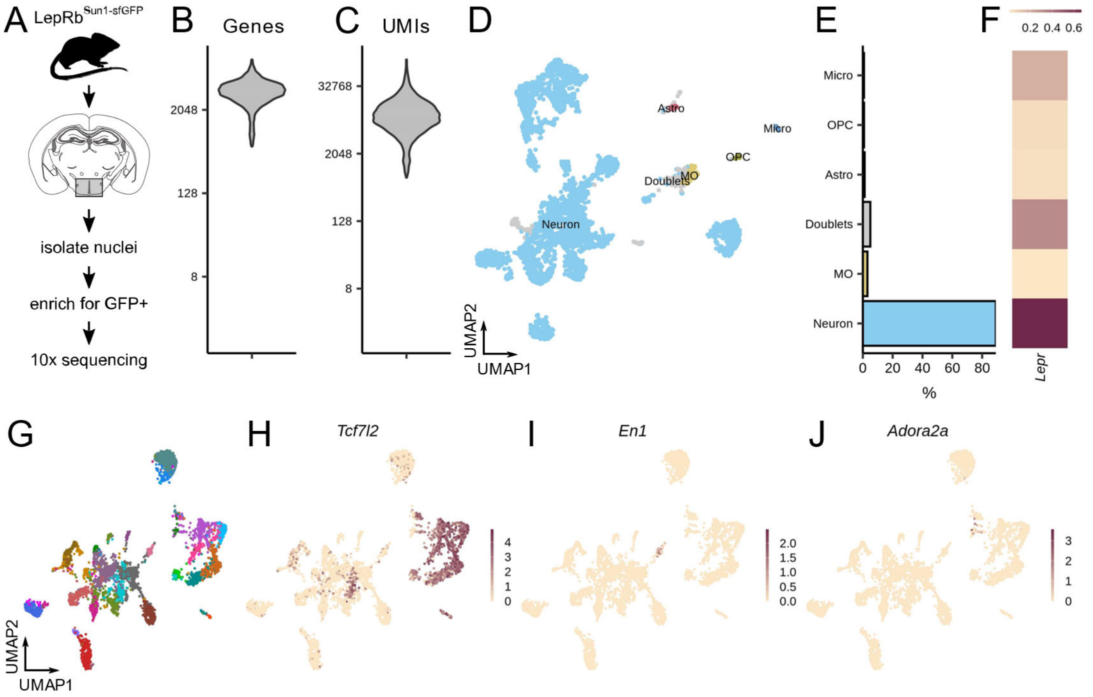

Supplemental Figure 2. Classification of nuclei from LepRb$^{Sun1\text{-}sfGFP}$ dataset. (**A**) Experimental design. (**B-C**) Distribution of genes (B) and UMIs (C) detected per nucleus. (**D**) UMAP projection of all cells colored by cell type. (**E**) Cell type membership breakdown. (**F**) Scaled *Lepr* expression in each cell type. Note that because nuclei contain predominantly unspliced RNA, it is not possible to determine which *Lepr* isoform is expressed by each cell type. (**G**) UMAP projections of all neurons, colored by cluster. (**H-J**) Expression of extrahypothalamic markers *Tcf7l2* (H), *En1* (I), and *Adora2a* (J) across all neurons.

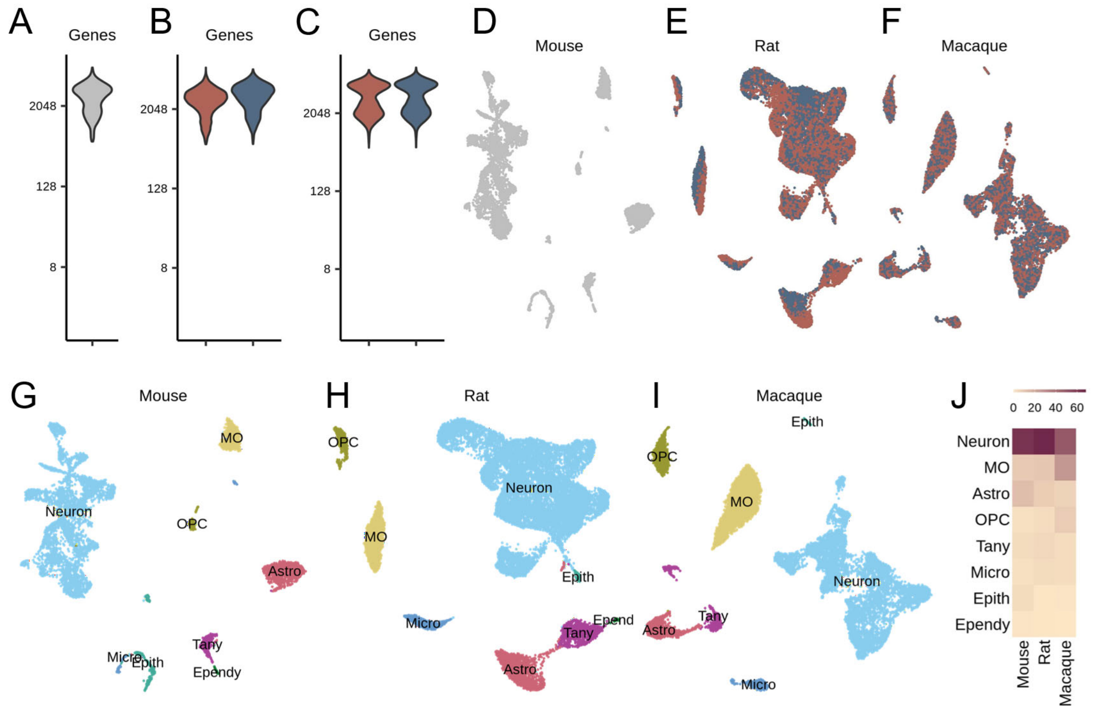

Supplemental Figure 3. Classification of nuclei from mouse, rat, and macaque datasets. (**A-C**) Distribution of genes per cells for the samples from mouse (~6300 cells) (A), rat (~16500 cells) (B), and macaque (C). (**D-F**) UMAP projection of all cells from mouse (D), rat (E), and macaque (F), colored by sample. (**G-I**) UMAP projection of all cells from mouse (G), rat (H), and macaque (I), colored by cell type. (**J**) Scaled *Lepr/LEPR* expression in each cell type for cells of each species. Note that because nuclei contain predominantly unspliced RNA, it is not possible to determine which *Lepr* isoform is expressed by each cell type.

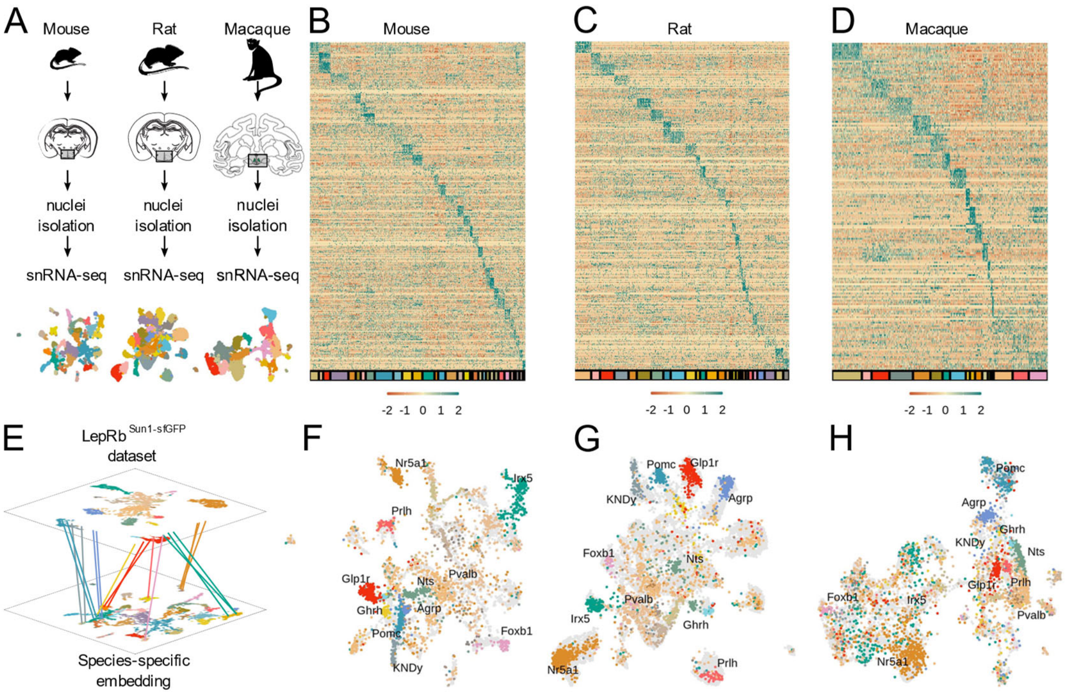

Supplemental Figure 4. Conservation of LepRb populations in rodents and macaques by projecting LepRb-Sun1 populations onto each species. (**A**) Diagram for snRNA-seq of mouse, rat, and macaque and UMAP projection of all neurons, colored by cluster. (**B**–**D**) Scaled expression of top marker genes for each species across all cells, colored by population (as in UMAP plots) on the bottom. (**E**) Strategy for identifying orthologous cells across datasets by projecting LepRb-Sun1 cells into UMAP space for hypothalamic cells from each species. (**F**–**H**) UMAP plot of each species (gray) overlayed with UMAP projections of LepRb-Sun1 cells (colors and labels are from LepRb-Sun1 populations as in Figure 1; labels are shown for populations that retain strong clustering across species).

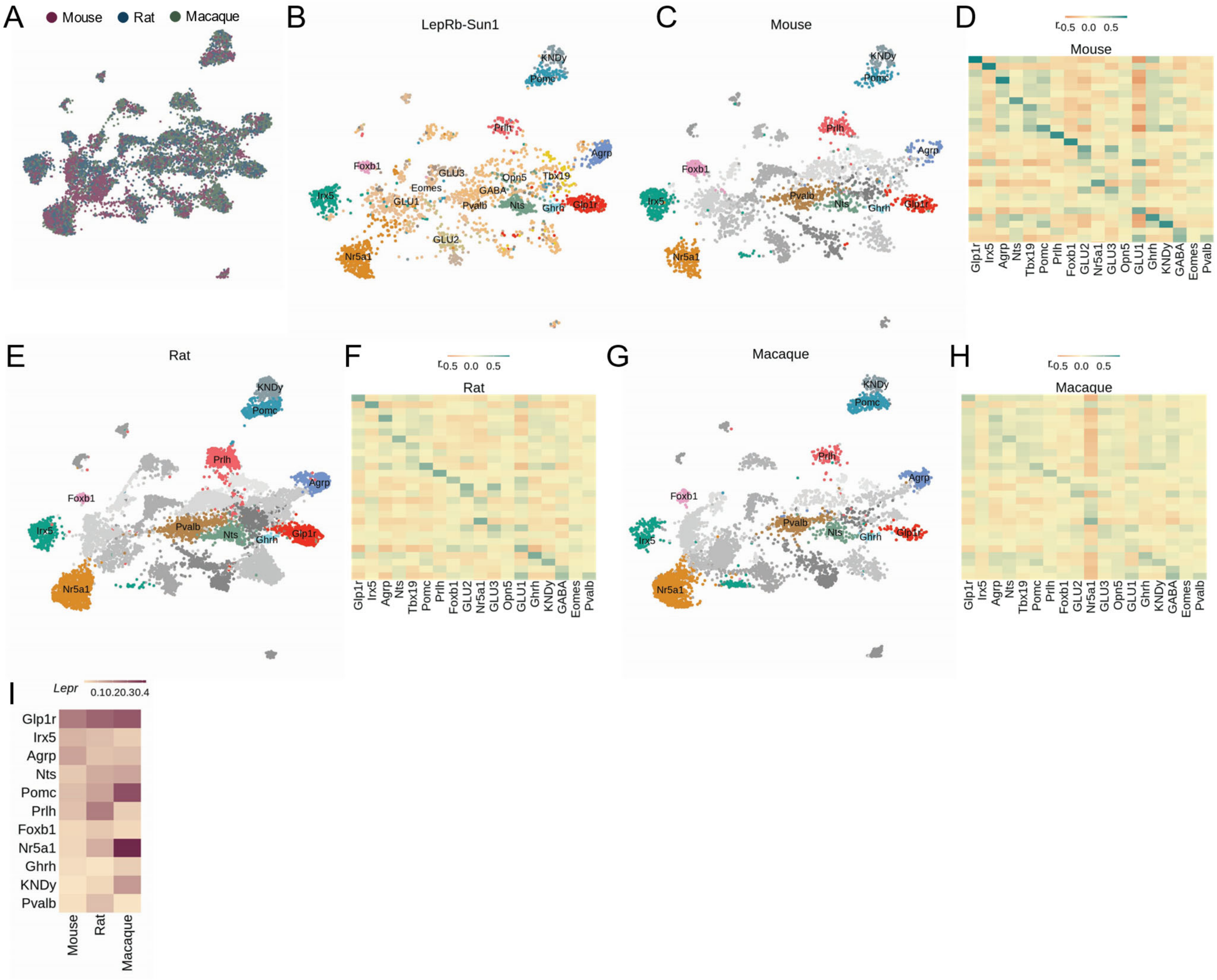

Supplemental Figure 5. Conservation of LepRb populations in rodents and macaques by co-clustering LepRb-Sun1 neurons with total hypothalamic neurons from mouse, rat and macaque. (**A**) UMAP plot of co-clustered cells from all species, using conserved orthologous genes; species of derivation of each cell is denoted by the color of the cell. (**B-H**) UMAP plots (B, C, E, G) and expression of cluster-specific marker genes (D, F, H) showing LepRb-Sun1 cells (B), mouse hypothalamic cells (C, D), rat hypothalamic cells (E, F), and macaque hypothalamic cells (G, H). Colors for marker genes represent scaled expression ranging from 0–1 for each species. (**I**) Mean *Lepr*/*LEPR* expression in each neuron populations from each species. Note that because nuclei contain predominantly unspliced RNA, it is not possible to determine which *Lepr* isoform is expressed by each cell type.

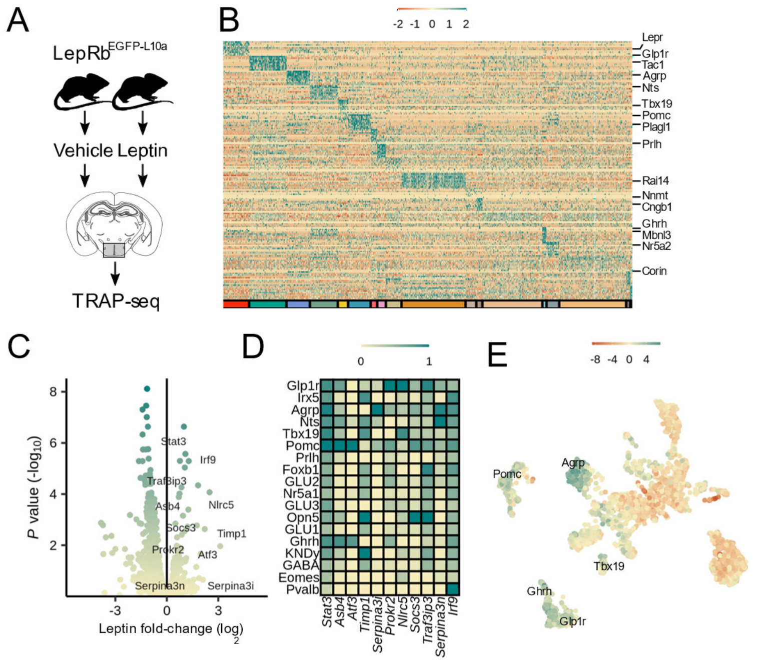

Supplemental Figure 6. Biased mapping of leptin-regulated transcripts toward specific LepRb-Sun1 populations. (**A**) Experimental diagram for published LepRb TRAP-seq (data available at GSE162603 (1)). (**B**) Relative expression of TRAP-seq enriched genes in specific LepRb-Sun1 populations. (**C**) Volcano plot of leptin-regulated changes in gene expression in TRAP-seq analysis. (**D**) Average expression of representative highly leptin-regulated genes across all LepRb-Sun1 clusters. (**E**) UMAP projection of all LepRb-Sun1 cells, colored by loading of leptin-regulated genes.

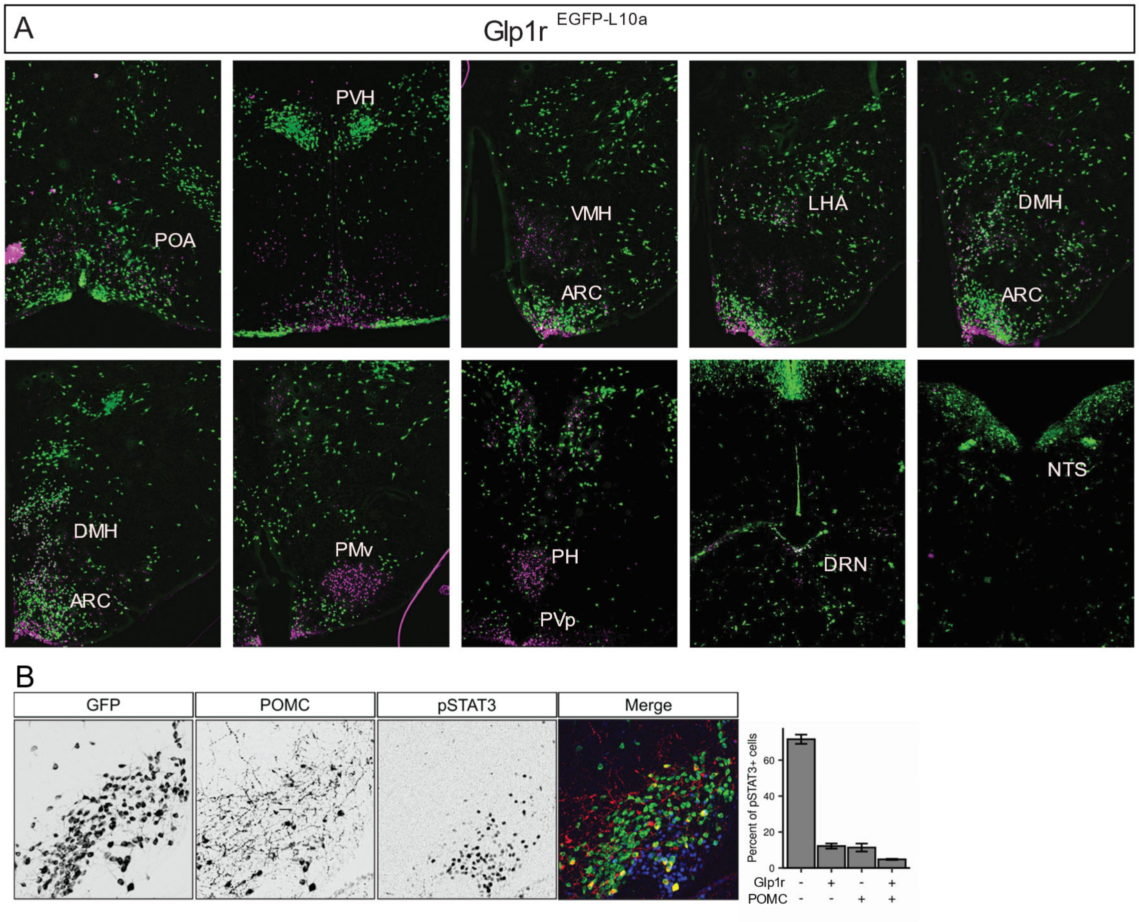

Supplemental Figure 7. Lack of LepRb(pSTAT3)/*Glp1r*(GFP) colocalization outside of the hypothalamus and lack of colocalization of LepRb^{Glp1r} neurons with POMC. **(A)** Representative images showing GFP-IR (green) and pSTAT3-IR (a marker for LepRb, magenta) from a leptin-treated Glp1r$^{EGFP\text{-}L10a}$ mouse, focusing on brain regions that exhibited staining for both. Note that the first panel of the bottom row also represents the image found in Figure 2C. **(B)** Representative image showing GFP-IR (left, green in merged panel), POMC-IR (second from left, red in merged panel), and pSTAT3-IR (third panel from left, blue in merged panel) from the ARC of a leptin-treated Glp1r$^{EGFP\text{-}L10a}$ mouse. Merged panel is the fourth panel from left. Right: Graph showing quantification of the percent of ARC pSTAT3-IR (LepRb) cells that colocalize with POMC-IR and/or GFP-IR (*Glp1r*). Mean -/+ SEM is shown, n=3.

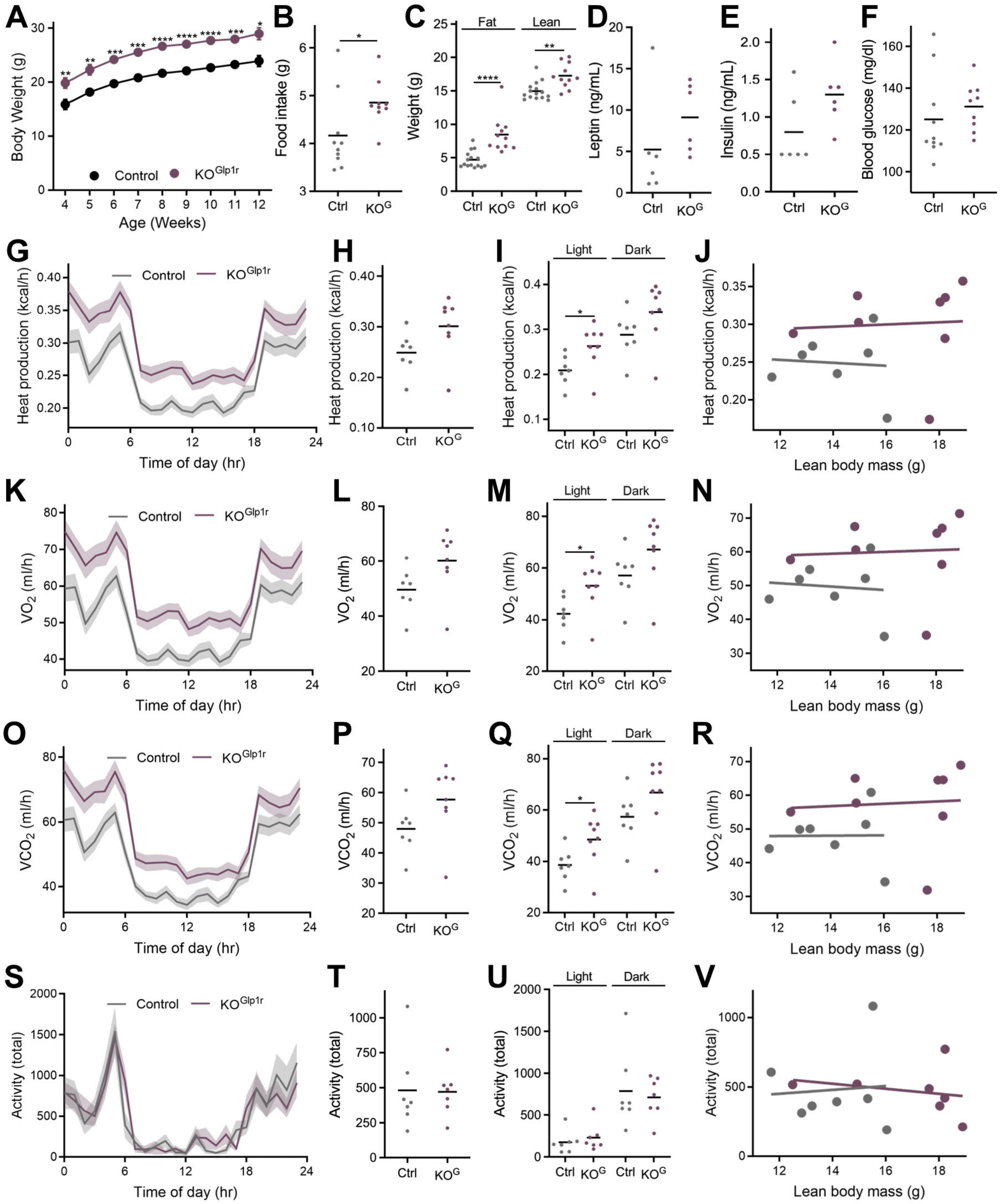

Supplemental Figure 8. Metabolic parameters in female KO^{Glp1r} mice. (**A-F**) Body weight (A), daily food intake (B), body composition (C), serum leptin (D), serum insulin (E), and blood glucose (F) (D-F: AM, *ad libitum*-fed) for female KO^{Glp1r} and Control mice. (**G-V**) measures of energy expenditure from metabolic cages studies in female KO^{Glp1r} and Control mice. Shown are Heat Production (G-J), VO_2 (K-N), VCO_2 (O-R), and Activity (S-V) across the diurnal cycle (G, K, O, S), over 24 hours (H, L, P, T), broken

down by light and dark cycles (I, M, Q, U), and plotted against lean body mass (J, N, R, V). *p<0.05; **p<0.01; ***p<0.001; ****p<0.0001 by Student's t-test.

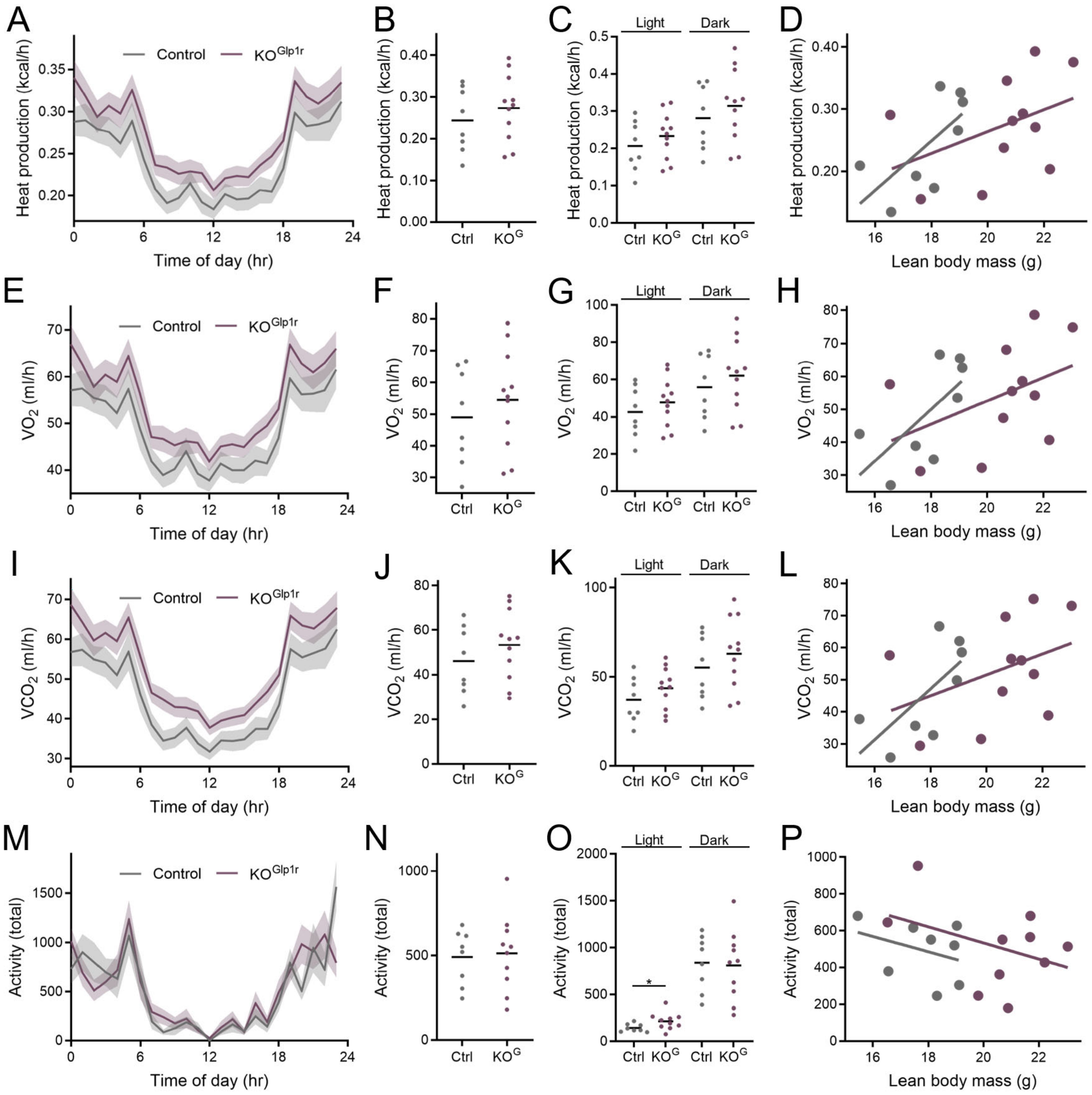

Supplemental Figure 9. Energy expenditure parameters in male KO^{Glp1r} mice. Shown are measures of energy expenditure from metabolic cages studies in male KO^{Glp1r} and Control mice: Heat Production (**A-D**), VO_2 (**E-H**), VCO_2 (**I-L**), and Activity (**M-P**) across the diurnal cycle (A, E, I, M), over 24 hours (B, F, J, N), broken down by light and dark cycles (C, G, K, O), and plotted against lean body mass (D, H, L, P). *$p<0.05$; **$p<0.01$; ***$p<0.001$; ****$p<0.0001$ by Student's t-test.

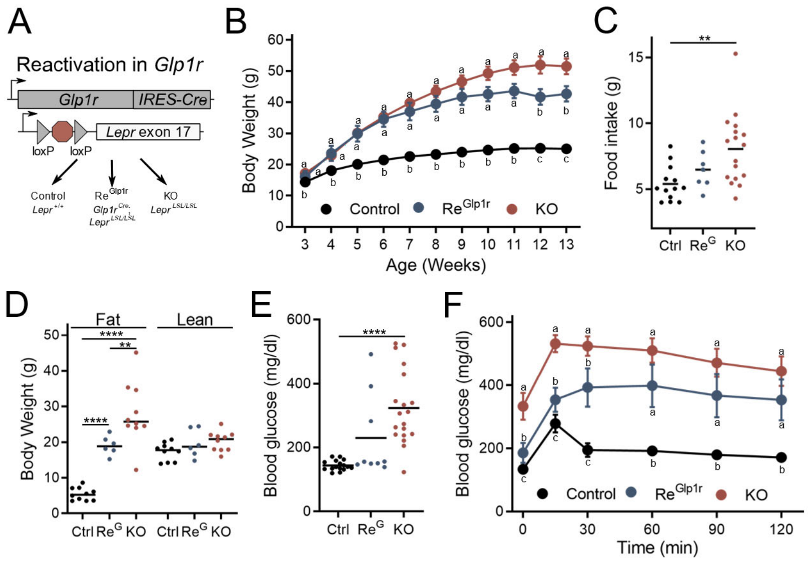

Supplemental Figure 10. Partial restoration of energy balance by *Lepr* expression in LepRb^{Glp1r} neurons in otherwise *Lepr*-null mice. (**A**) Experimental strategy showing Control, *Glp1r^{Cre};Lepr$^{LSL/LSL}$* (Re^{Glp1r} or Re^{G}) and *Lepr$^{LSL/LSL}$* (KO) animals. (**B-F**) Body weight (B), food intake (C), body composition (D), *ad libitum*-fed AM blood glucose (E), and glycemic response to an intraperitoneal glucose tolerance test (F) in mixed sex Control (black), Re^{Glp1r} (blue), and KO (red) mice. Panels B, F show mean -/+ SEM; different letters signify conditions that are statistically different ($p<0.05$) by ANOVA with Tukey's post-hoc test. All other panels: *$p<0.05$; **$p<0.01$; ***$p<0.001$; ****$p<0.0001$ by ANOVA with Dunnett's post-hoc test.

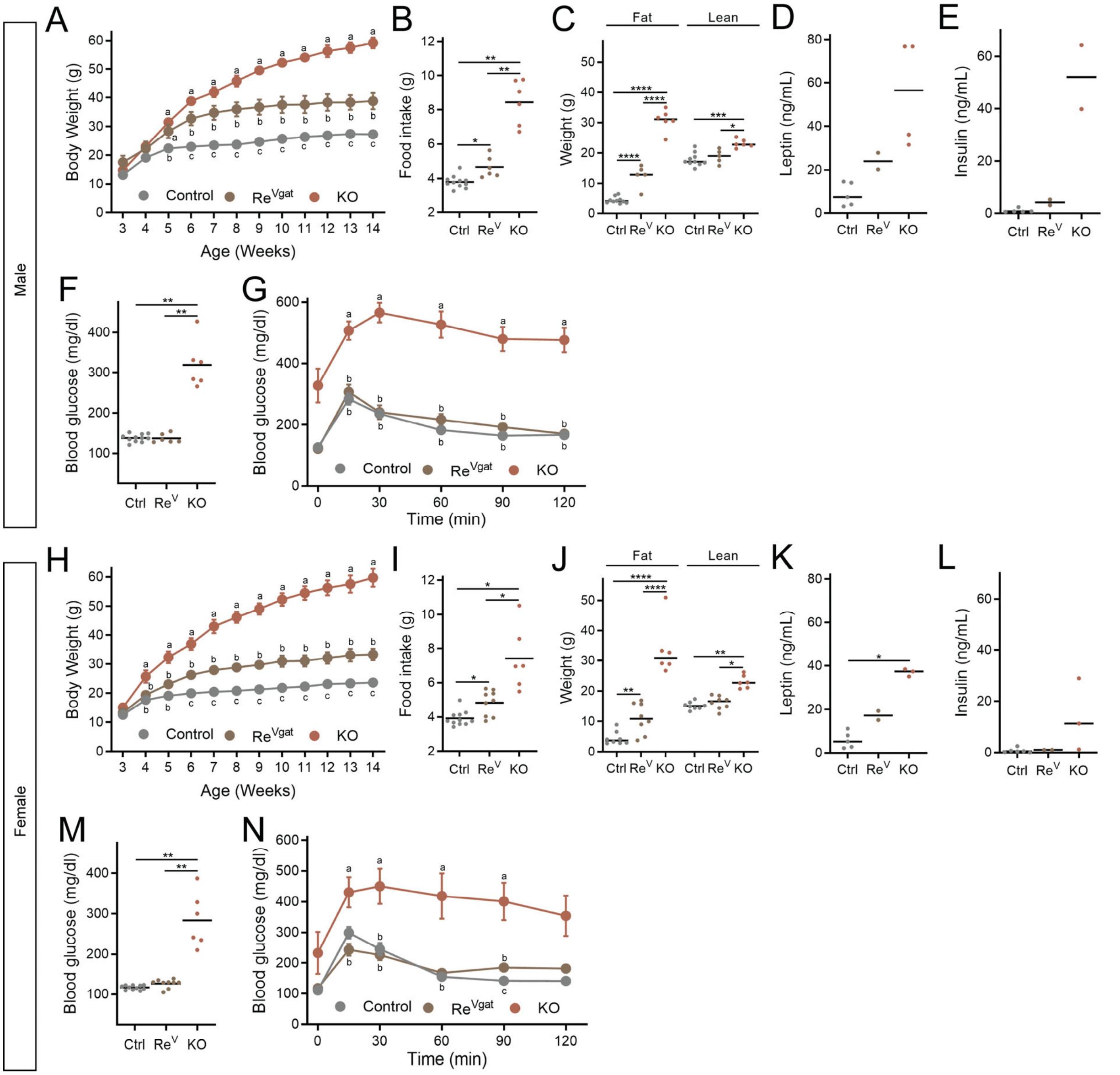

Supplemental Figure 11. Data from Figure 4, separated by sex. For Males (**A-G**): Body weight (A; n=11 Ctrl, 6 Re^{V}, 6 KO male), food intake (B; n=11, 6, 6), body composition (C; n=10, 5, 6), serum leptin (D; n=5, 2, 4), serum insulin (E; n= 5, 2, 2), blood glucose (F; n=11, 6, 6) (D-F all AM, *ad libitum*-fed), and glycemic response to intraperitoneal glucose tolerance test (G) in control (gray; n= 10 male), KO (orange; n= 4), and Re^{Vgat} (gold; n= 6) mice. For Females (**H-N**): Body weight (H; n= 11 Ctrl, 9 Re^{V}, 6 KO female), food intake (**I**; n= 11, 9, 6), body composition (J; n= 8, 8, 6), serum leptin (K; n= 5, 2, 3), serum insulin (L; n=6, 3, 2), blood glucose (M; n= 11, 9, 6) (K-M all AM, *ad libitum*-fed), and glycemic response to intraperitoneal glucose tolerance test (N) in control (gray; n= 9 female), KO (orange; n=5), and Re^{Vgat} (gold; n=9) mice. Panels A, G, H, N show mean -/+ SEM; different letters signify conditions that are statistically

different ($p<0.05$) by ANOVA with Tukey's post-hoc test. All other panels: *$p<0.05$; **$p<0.01$; ***$p<0.001$; ****$p<0.0001$ by ANOVA with Dunnett's post-hoc test.

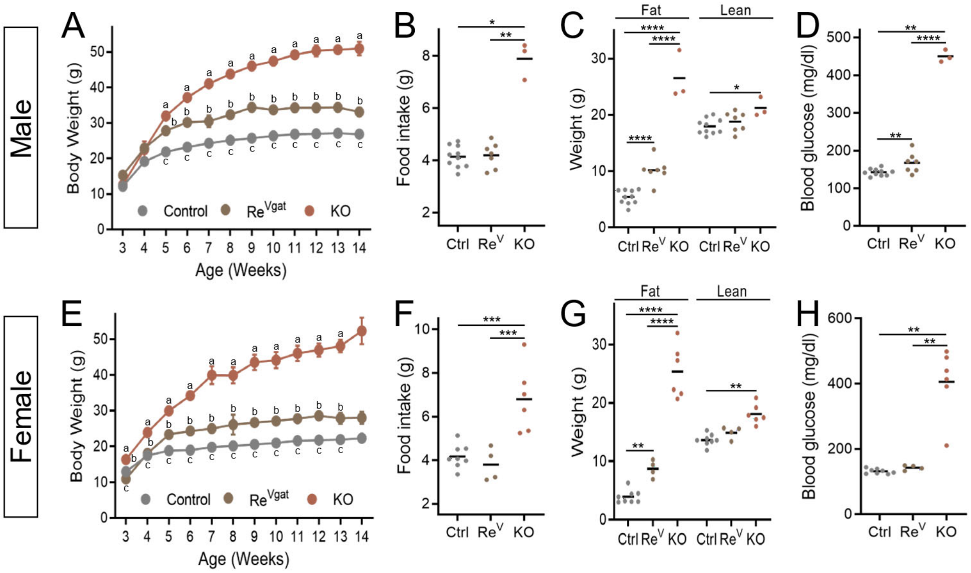

Supplemental Figure 12. Requirement for *Lepr* in GABAergic *Glp1r* cells for the control of energy balance. Shown are data for male (A-D) and female (E-H) Control (gray), *Lepr*$^{LSL/LSL}$ (KO, red), *Scl32a1*^{Cre};*Lepr*$^{LSL/LSL}$ (Re^{Vgat}, brown) mice. (**A, E**) Body weight, (**B, F**) food intake, (**C, G**) body composition, and (**D, H**) blood glucose (AM *ad libitum*-fed). For panels A, E mean -/+ SEM are plotted; different letters signify conditions that are statistically different ($p<0.05$) by ANOVA with Tukey's post-hoc test. All other panels: *$p<0.05$; **$p<0.01$; ***$p<0.001$; ****$p<0.0001$ by ANOVA with Dunnett's post-hoc test.

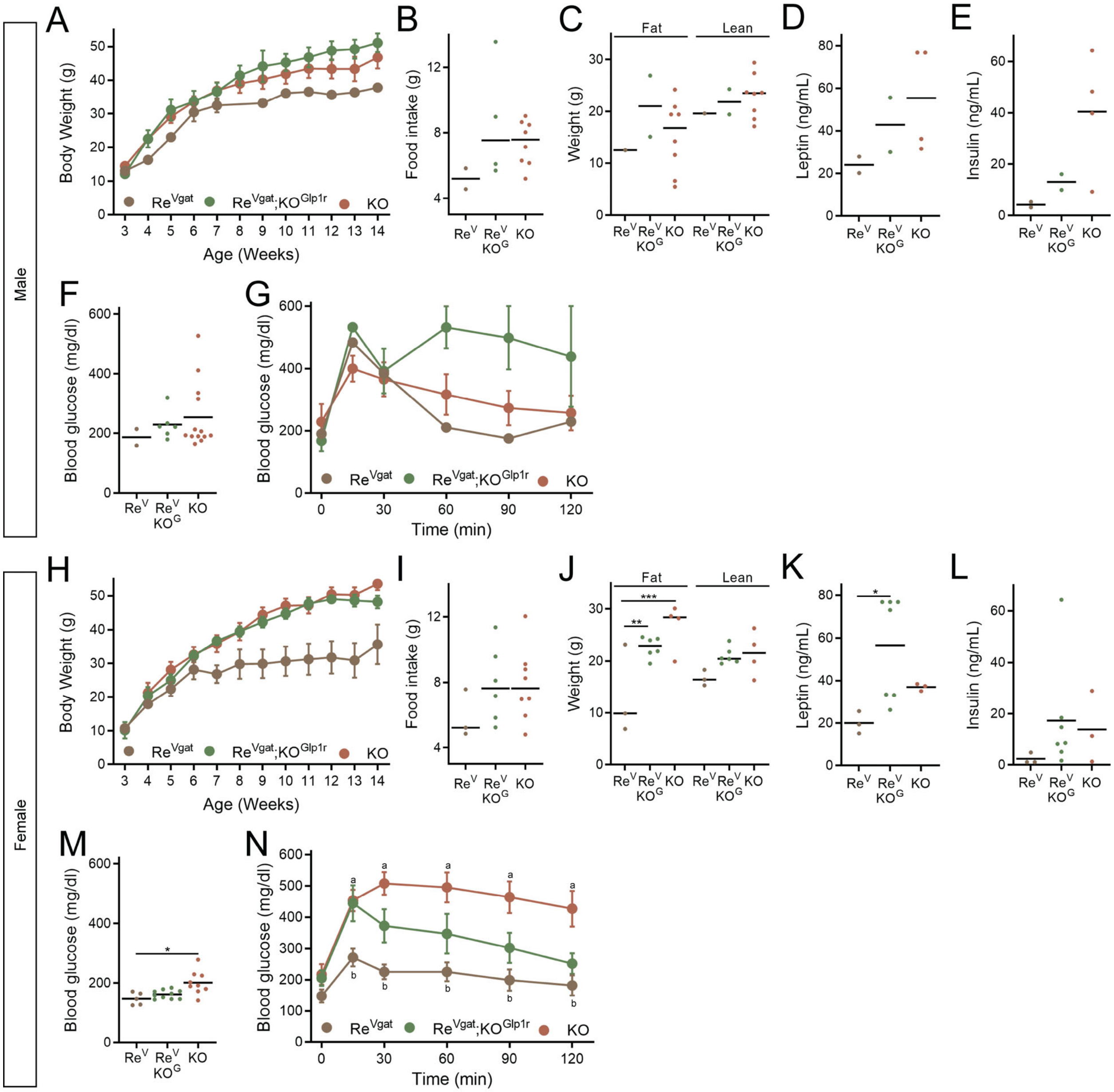

Supplemental Figure 13. Data from Figure 5, separated by sex. For Males (**B-H**): Body weight (A; n= 2 Re^{V}, 6 Re^{V}KO^{G}, and 13 KO), food intake (B; n= 2, 3, 8), body composition (C; n= 1, 2, 8), serum leptin and serum insulin (D,E; n=2, 2, 4), blood glucose (F; n= 2, 6, 13) (D-F all AM *ad libitum*-fed), and glycemic response to intraperitoneal glucose tolerance test (G) in Re^{vGAT} (gold; n= 1), KO (orange; n= 8), and Re^{vGAT}KO^{Glp1r} (green; n= 2) mice. For Females (**H-N**): Body weight (H; n= 5 Re^{V}, 10 Re^{V}KO^{G}, 9 KO), food intake (I; n= 3, 7, 8), body composition (J; n= 3, 6, 4), serum leptin and serum insulin (K, L; n= 3, 7, 3), blood glucose (M; n= 5, 10, 9) (K-M all AM *ad libitum*-fed), and glycemic response to intraperitoneal glucose tolerance test (N) in Re^{vGAT} (gold; n= 5), KO (orange; n= 5), and Re^{vGAT}KO^{Glp1r} (green; n= 6 females) mice. For panels A, G, H, N show mean -/+ SEM; different letters signify conditions that are

statistically different ($p<0.05$) by ANOVA with Tukey's post-hoc test. All other panels: *$p<0.05$; **$p<0.01$; ***$p<0.001$; ****$p<0.0001$ by ANOVA with Dunnett's post-hoc test.

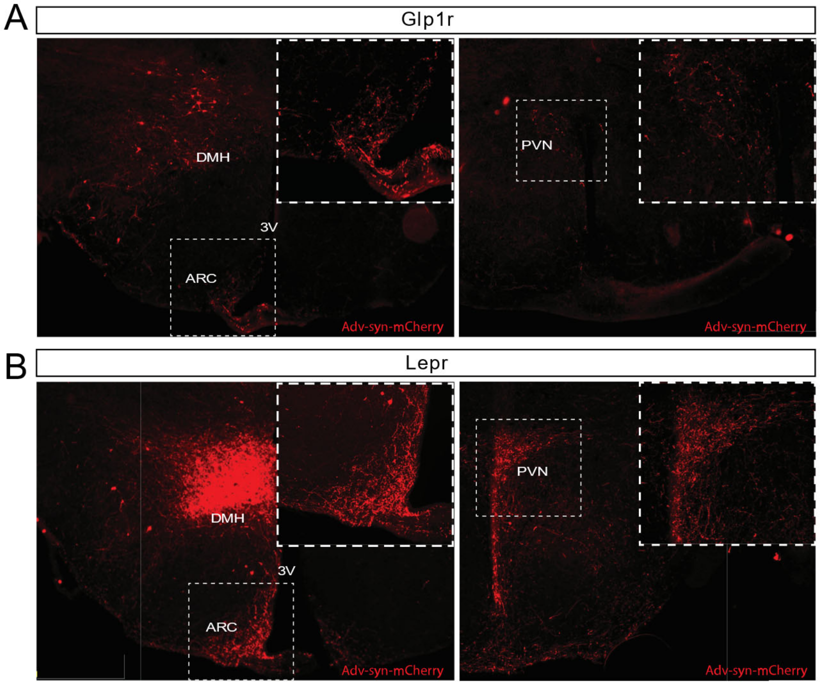

Supplemental Figure 14. Projections from DMH *Glp1r*- and *Lepr*-expressing neurons. *Glp1r*^{Cre} (**A**) and *Lepr*^{Cre} (**B**) mice were injected in the DMH with the cre-dependent AdV-iN-Synaptophysin-mCherry (Adv-syn-mCherry)(2) and perfused 5 days later. Brains were sectioned and stained for dsRed (mCherry, red). Representative images show the injection site and ARC (right panels) and PVH (right panels)- the main projection targets observed for DMH *Glp1r* (A) and DMH *Lepr* (B) neurons.

1. Allison MB, Pan W, Mackenzie A, Patterson C, Shah K, Barnes TM, Cheng W, Rupp A, Olson DP, and Myers MG. Defining the transcriptional targets of leptin reveals a role for Atf3 in leptin action. *Diabetes.* 2018;In Press(
2. Leinninger GM, Jo YH, Leshan RL, Louis GW, Yang H, Barrera JG, Wilson H, Opland DM, Faouzi MA, Gong Y, et al. Leptin acts via leptin receptor-expressing lateral hypothalamic neurons to modulate the mesolimbic dopamine system and suppress feeding. *Cell Metab.* 2009;10(2):89-98.
